# Optimal localization of diffusion sources in complex networks

**DOI:** 10.1098/rsos.170091

**Published:** 2017-04-12

**Authors:** Zhao-Long Hu, Xiao Han, Ying-Cheng Lai, Wen-Xu Wang

**Affiliations:** 1 School of Systems Science, Beijing Normal University, Beijing 100875, People’s Republic of China; 2 School of Electrical, Computer and Energy Engineering, Arizona State University, Tempe, AZ 85287, USA; 3 Department of Physics, Arizona State University, Tempe, AZ 85287, USA; 4 Business School, University of Shanghai for Science and Technology, Shanghai 200093, People’s Republic of China

**Keywords:** diffusion sources, optimal localization, complex networks, locatability, compressive sensing

## Abstract

Locating sources of diffusion and spreading from minimum data is a significant problem in network science with great applied values to the society. However, a general theoretical framework dealing with optimal source localization is lacking. Combining the controllability theory for complex networks and compressive sensing, we develop a framework with high efficiency and robustness for optimal source localization in arbitrary weighted networks with arbitrary distribution of sources. We offer a minimum output analysis to quantify the source locatability through a minimal number of messenger nodes that produce sufficient measurement for fully locating the sources. When the minimum messenger nodes are discerned, the problem of optimal source localization becomes one of sparse signal reconstruction, which can be solved using compressive sensing. Application of our framework to model and empirical networks demonstrates that sources in homogeneous and denser networks are more readily to be located. A surprising finding is that, for a connected undirected network with random link weights and weak noise, a single messenger node is sufficient for locating any number of sources. The framework deepens our understanding of the network source localization problem and offers efficient tools with broad applications.

## 1. Introduction

Dynamical processes taking place in complex networks are ubiquitous in natural and in technological systems [1], examples of which include disease or epidemic spreading in the human society [2,3], virus invasion in computer and mobile phone networks [4,5], behaviour propagation in online social networks [6] and air or water pollution diffusion [7,8]. Once an epidemic or environmental pollution emerges, it is often of great interest to be able to identify its source within the network accurately and quickly so that proper control strategies can be devised to contain or even to eliminate the spreading process. In general, various types of spreading dynamics can be regarded as diffusion processes in complex networks, and it is of fundamental interest to be able to locate the *sources of diffusion*. A straightforward, brute-force search for the sources requires accessibility of global information about the dynamical states of the network. However, for large networks, a practical challenge is that our ability to obtain and process global information can often be quite limited, making brute-force search impractical with undesired or even disastrous consequences. For example, the standard breadth-first search algorithm for finding the shortest paths, when being implemented in online social networks, can induce information explosion even for a small number of searching steps [9]. Recently, in order to locate the source of the outbreak of Ebola virus in Africa, five medical practitioners lost their lives [10]. All these call for the development of efficient methodologies to locate diffusion sources based only on limited, practically available information without the need of acquiring global information about the dynamical states of the entire network.

There were pioneering efforts in addressing the source localization problem in complex networks, such as those based on the maximum-likelihood estimation [11], belief propagation [12], the phenomena of hidden geometry of contagion [13] and inverse spreading [14,15]. In addition, some approaches have been developed for identifying super spreaders that promote spreading processes stemming from sources [16–18]. In spite of these efforts, achieving accurate source localization from a small number of measurements remains challenging. Prior to our work, a systematic framework dealing with the localization of diffusion sources for arbitrary network structures and interaction strength was missing.

In this paper, we develop a theoretical framework to address the problem of network source localization in a detailed and comprehensive way. The main focus is on the fundamental issue of *locatability*, i.e. given a complex network and limited (sparse) observation, are diffusion sources locatable? A practical and extremely challenging issue is, given a network, can a minimum set of nodes be identified which produce sufficient observation so that sources at arbitrary locations in the network can actually be located? To address these issues in a systematic manner, we use a two-step solution strategy. First, we develop a minimum output analysis to identify the minimum number of messenger/sensor nodes, denoted as *N*_m_, to fully locate any number of sources in an efficient way. The ratio of *N*_m_ to the network size *N*, *n*_m_≡*N*_m_/*N*, thus characterizes the source locatability of the network in the sense that networks requiring smaller values of *n*_m_ are deemed to have a stronger locatability of sources. Our success in offering the minimum output analysis stems from taking advantage of the dual relation between the recently developed controllability theory [19] and the canonical observability theory [20]. Second, given *N*_m_ messenger nodes, we formulate the source localization problem as a sparse signal reconstruction problem, which can be solved by using compressive sensing (CS) [21,22], a convex optimization paradigm. The basic properties of CS allow us to accurately locate sources from a small amount of measurement from the messenger nodes, much less than that required in the conventional observability theory. We use our framework to examine a variety of model and real-world networks, and offer analytical prediction of *n*_m_ and demonstrate good agreement with numerical calculations. We find that the connection density and degree distribution play a significant role in source locatability, and sources in a homogeneous and denser network are more readily to be located, which differs from existing algorithms for source localization in the literature [11,14,15]. A striking and counter-intuitive finding is that, for an undirected network with one connected component and random link weights, a single messenger node is sufficient to locate any number of sources in the presence of weak noise.

Theoretically, the combination of the minimum output analysis (derived from the controllability and observability theories for complex networks) and the CS-based localization method constitutes a general framework for locating diffusion sources in complex networks. It represents a powerful paradigm to exactly quantify the source locatability of a network and to actually locate the sources efficiently and accurately. Because of the CS-based methodology, our framework is robust against noise [23,24], paving way to practical implementation in noise environment.

## 2. Results

### 2.1. A general framework to locate sources with minimum number of messenger nodes

We consider a class of diffusive processes on networks, described by

2.1 $$x_{i}(t+1)=x_{i}(t)+\beta \sum_{j=1}^{N}[w_{ij}x_{j}(t)-w_{ji}x_{i}(t)].$$

This equation constitutes a good approximation for different types of linear diffusion processes and the linearization of some nonlinear diffusion processes [25]. For example, epidemics can be treated as linear dynamics in the early stages if the network connectivity is high. Variable *x*_*i*_(*t*) that denotes the state of node *i* at time *t* captures the fraction of infected individuals, the concentration of water or air pollutant, etc., at place *i*. *β* is the diffusion coefficient, *w*_*ij*_ (*w*_*ji*_) is the weight of the directed link from node *j* to node *i* (*i* to *j*), (*w*_*ij*_=*w*_*ji*_ for undirected networks), and *N* is the number of nodes in the network (size). It is noteworthy that the value of the diffusion parameter *β* should be constrained to ensure the physical meaning of *x*_*i*_(*t*), i.e. *x*_*i*_(*t*) is confined in the range [0,1] at any time *t* for any node. We can prove that the confinement of *x*_*i*_(*t*) leads to $\beta \in (0,\min_{i=1,2,\ldots ,N}(1/\sum_{j=1,\;j\neq i}^{N}w_{ji})]$ (see electronic supplemental material, S1 for the proof). Equation (2.1) is discrete in time, greatly facilitating computation and analysis. When observations are made from a subset of nodes, the messenger nodes, system (2.1) incorporating outputs from these nodes can be written concisely as

**Figure 1.**
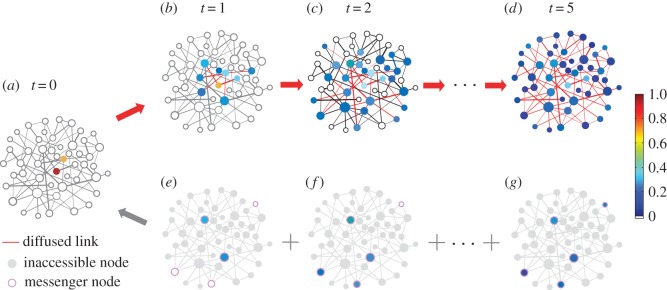
Illustration of source localization problem. (*a*) A random network with two sources at the initial time *t*=0. (*b*–*d*) The diffusion process at *t*=1 (*b*), *t*=2 (*c*) and *t*=5 (*d*), respectively. The colour bar represents the state of node *x*_*i*_(*t*), and those links along which diffusion occurred are marked with red. Panels (*a*) to (*d*) describe a diffusion (spreading) process from two sources to the whole network according to equation (2.1). (*e*–*g*) Five messenger nodes whose states at three time constants can be measured and collected. The messenger nodes are specified by the output matrix *C* and the states of messenger nodes and inaccessible nodes constitute **y**(*t*). The time of (*e*), (*f*) and (*g*) corresponds to (*b*), (*c*) and (*d*), respectively. However, in the real situation, the time as well as the initial time is unknown. The only available information for locating sources is the states of a set of messenger nodes at some time and the network structure. (*e*), (*f*) and (*g*) to (*a*) describe the source localization problem to be solved. Moreover, we aim to identify a minimum set of messenger nodes to locate an arbitrary number of sources at any location by virtue of our minimum output analysis and optimization based on compressive sensing.

2.2 $$\left\{ \begin{array}{l} \text{x}(t+1)=(I+\beta L)\text{x}(t),\\ \text{y}(t)=C\text{x}(t), \end{array}\right.$$

where $\text{x}(t)\in R^{N}$ is the state vector of the entire network at time *t*, $I\in R^{N\times N}$ is the identity matrix, *L*=(*W*−*D*) is a Laplacian matrix, $W\in R^{N\times N}$ is the weighted adjacency matrix of elements *w*_*ij*_, $D\in R^{N\times N}$ is a diagonal matrix of elements *d*_*i*_ denoting the total out-weight $\sum_{j\in \Gamma_{i}}w_{ji}$ of node *i*, where *Γ*_*i*_ is the neighbouring set of *i*. The vector $\text{y}(t)\in R^{q}$ is the output at time *t* and $C\in R^{q\times N}$ is the *output matrix*. Messenger nodes are specified through matrix *C* and **y**(*t*) records the states of these nodes. The source localization problem is illustrated in figure 1, which is a kind of inverse problem for diffusion and spreading dynamics on complex networks.

The basic difference between source nodes and other nodes in the network is that initially (*t*=*t*_0_), the states of the former are non-zero while those of the latter are zero. To achieve accurate localization of an arbitrary number of sources at arbitrary locations, it is only necessary to recover the initial states of all nodes from the measurements of the messenger nodes at a later time (*t*>*t*_0_). A solution to this problem can be obtained using the observability condition in canonical control theory. To be specific, we consider instants of time: *t*_0_,*t*_1_,…,*t*, and perform a simple iterative process that yields the relation between **x**(*t*) and **x**(*t*_0_): **x**(*t*)=[*I*+*βL*]^*t*−*t*_0_^**x**(*t*_0_). Consequently, the output, which depends on **x**(*t*_0_), can be expressed as **y**(*t*)=*C*(*I*+*βL*)^*t*−*t*_0_^**x**(*t*_0_). The key to accurate localization of sources lies in the existence of a unique solution of the equation, given the output vector **y**(*t*) from the set of messenger nodes as specified by *C*. Intuitively, to obtain a unique solution, no fewer than *N* snapshots of measurement are needed. Without loss of generality, we assume that uninterrupted time series from *t*_0_ to *t*_0_+*N*−1 are available. We obtain

2.3 $$\text{Y}=O\cdot \text{x}(t_{0}),$$

where $\text{Y}\in R^{qN}$, the initial state vector is $\text{x}(t_{0})\in R^{N}$, *q* is the number of messenger nodes, and the matrix $O\in R^{qN\times N}$ is nothing but the observability matrix in the canonical control theory (see §5.1 for details of equation (2.3)). The observability full rank condition [26] stipulates that, if and only if rank(*O*)=*N*, there exists a unique solution of equation (2.3) and the state vector **x**(*t*_0_) at initial time *t*_0_ is observable. Insofar as the given output matrix *C* satisfies the observability rank condition, the initial states of the nodes can be fully reconstructed from the states of the messenger nodes, and all sources can then be located. A challenge is that, in a realistic situation, the initial time *t*_0_ is often unknown, rendering the immediate application of the canonical observability condition invalid. However, a unique and desired feature of our framework is that both **x**(*t*_0_) and *t*_0_ can be inferred based on CS (see §§3 and 5.2). Thus, it is possible to develop a theoretical framework on the basis of the observability condition (see electronic supplementary material, S2 for continuous-time processes).

### 2.2. Minimum number of messengers for source localization

Beyond the canonical observability theory, here our goal is to identify a minimum set of messenger nodes to satisfy the full rank condition for observability. However, the brute-force method of enumerating all possible choices of the messenger nodes is computationally prohibitive [27], as the total number of possible configurations is 2^*N*^. Our solution is to use the recently developed, exact controllability framework [19] based on the standard Popov–Belevitch–Hautus (PBH) test theory [28] and to exploit the dual relationship between controllability and observability [20], which results in a practical framework to find the required *N*_m_ messenger nodes. In particular, for an arbitrary network, according to the PBH test and the exact controllability framework, *N*_m_ is determined by the maximum geometric multiplicity of the eigenvalues λ_*i*_ of the matrix *I*+*βL*. After some matrix calculation, we obtain that (see electronic supplementary material, S3)

2.4 $$N_{m}=\max_{i}\{N-\mathrm{rank}[\lambda_{i}^{L}I-L]\},$$

where $\lambda_{i}^{L}$ is the eigenvalue of matrix *L* and $\mu (\lambda_{i}^{L})\equiv N-\mathrm{rank}[\lambda_{i}^{L}I-L]$ is the geometric multiplicity of $\lambda_{i}^{L}$. It is worth noting that the formula of *N*_m_ does not contain the diffusion parameter *β*, indicating that choices of *β* do not affect the locatability measure *n*_m_. Equation (2.4) as a result of the standard PBH test is a general minimum output analysis for arbitrary networks.

For an undirected network, *L* is symmetric and the geometric multiplicity is nothing but the eigenvalue degeneracy. In addition, the eigenvalue degeneracy of *L* is equal to that of *I*+*βL* (see electronic supplementary material, S3). Thus, *N*_m_ is determined by the maximum eigenvalue degeneracy of *L* as

2.5 $$N_{m}^{\mathrm{undirect}}=\max_{i}\{\delta (\lambda_{i}^{L})\},$$

where $\delta (\lambda_{i}^{L})$ is the degeneracy of $\lambda_{i}^{L}$ (the number of appearances of $\lambda_{i}^{L}$ in the eigenvalue spectrum). Equation (2.5) based on the PBH test is our minimum output analysis for arbitrary undirected networks.

Equations (2.4) and (2.5) are the exact theory (ET) for minimum output *N*_m_ without any approximations, but the associated computational cost resulting from calculating the eigenvalues and identifying maximum value through a large number of comparisons in equations (2.4) and (2.5) is generally high. Taking advantage of the ubiquitous sparsity of real networks [29], we can obtain an alternative method to estimate *N*_m_ with much higher efficiency. In particular, for sparse networks, we have (see electronic supplementary material, S4)

2.6 $$n_{m}^{\mathrm{sparse}}\approx 1-\frac{\mathrm{rank}(aI-L)}{N},$$

where for undirected networks, *a* is either zero or the diagonal element with the maximum multiplicity (number of appearances in the diagonal) of matrix *L*. The matrix rank as well as eigenvalues in formula (2.6) can be computed using fast algorithms from computational linear algebra, such as SVD with the computation complexity *O*(*N*^3^) [30] or LU decomposition with the computation complexity *O*(*N*^2.376^) [31]. In general, equation (2.6) allows us to compute *n*_m_ efficiently, thereby the term *fast estimation* (FE) method.

### 2.3. Analytical results for model networks

**Figure 2.**
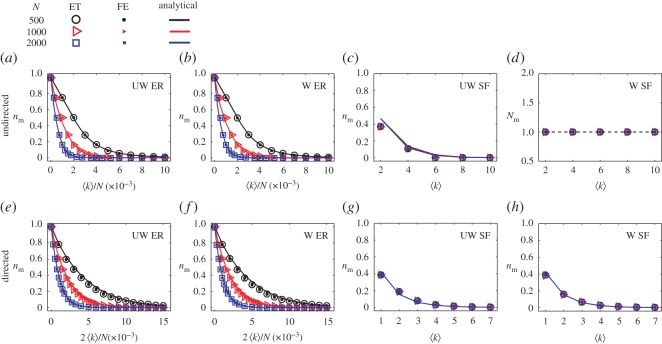
Locatability measure *n*_m_ for ER and SF networks. (*a*–*b*) For undirected networks, source locatability measure *n*_m_ as a function of the connecting probability 〈*k*〉/*N* for (*a*) unweighted ER networks and (*b*) weighted ER networks. (*c*–*d*) *n*_m_ as a function of the average degree 〈*k*〉 for (*c*) unweighted SF networks, and *N*_m_ as a function of the average degree 〈*k*〉 for (*d*) weighted SF networks. For undirected networks, the values of *n*_m_ are obtained from the exact theory (ET; equation (2.5)), fast estimation (FE; equation (2.6)), and analytical prediction (Analytical), for different network sizes. The analytical prediction for ER networks is based on equation (2.7). For SF networks in (*c*), the prediction is from the cavity method. (*e*–*h*) For directed networks, source locatability measures *n*_m_ as a function of the connecting probability 2〈*k*〉/*N* for (*e*) unweighted and (*f*) weighted ER networks, and as a function of 〈*k*〉 for (*g*) unweighted and (*h*) weighted SF networks. For directed networks, the ET results come from equation (2.4), while the FE results for ER and SF networks are from equation (2.6). The analytical predictions for ER and SF networks are from equations (2.8) and (2.9), respectively. For weighted networks, link weights are randomly selected from a uniform distribution in the range (0,2), which leads to that the mean weight is approximately one. The ET and FE results are obtained by averaging over 50 independent realizations, and the error bars represent the standard deviations. For undirected ER networks, 〈*k*〉 = *Np*_con_, where *p*_con_ is the connecting probability between each pair of nodes. Thus, *p*_con_=〈*k*〉/*N*. For directed ER networks, 〈*k*〉 = *Np*_con_/2, yielding *p*_con_=2〈*k*〉/*N*.

We first apply our minimum output analysis to undirected Erdös–Rényi (ER) random [32] and scale-free (SF) [33] networks and derive analytical results. Figure 2 shows that, as the average degree 〈*k*〉 ($\langle k\rangle \equiv (1/N)\sum_{i}^{N}k_{i}$, where *k*_*i*_ is the node degree of *i*) is increased, *n*_m_ decreases for undirected ER random networks with identical and random link weights. For the random networks, the efficient formula (2.6) can be further simplified. In particular, for small values of 〈*k*〉, due to the isolated nodes and the disconnected components, zero dominates the eigenvalue spectrum of the matrix *L* [34] where, for example, each disconnected component generates at least one zero eigenvalue in *L*. For large values of 〈*k*〉, we expect all eigenvalues to be distinct without any dominant one. In this case, we can still choose zero to be the eigenvalue associated with *a* in equation (2.6). Taken together, in a wide range of 〈*k*〉 values, the efficient formula equation (2.6) holds with *a*=0. Alternatively, the value of *n*_m_ for ER networks can be theoretically estimated using the degree distribution because of the dominance of the null eigenvalue (see electronic supplementary material, S4)

2.7 $$n_{m}^{\mathrm{UER}}\approx \left\{ \begin{array}{ll} 1-\langle k\rangle /2 & \left\langle k\rangle \in [0,1\right]\\ \frac{1}{\left\langle k\right\rangle }(f(\langle k\rangle )-f{\left(\langle k\rangle \right)}^{2}/2) & \langle k\rangle \in (1,\infty ), \end{array}\right.$$

where $f(\langle k\rangle )=\sum_{k=1}^{\infty }(k^{k-1}/k!){\left(\langle k\rangle e^{-\langle k\rangle }\right)}^{k}$.

For undirected SF networks, *a* in the efficient formula (2.6) is the diagonal element with the maximum number of appearances in the diagonal of matrix *L*. In the controllability framework, the density of the driver nodes can be calculated [34,35] with the cavity method [36]. The principle can be extended to analysing locatability measure of SF networks in a similar manner (see electronic supplementary material, S5). The analytical estimation for both ER and SF networks is in good agreement with the results of ET and FE, as shown in figures 2*a*–*d*. Indeed, the results indicate that choosing *a*=0 in the efficient formula (2.6) is justified for the ER networks. For small values of 〈*k*〉, zero dominates the eigenvalue spectrum, and there are a number of messenger nodes with *n*_m_>1/*N*. When 〈*k*〉 exceeds a certain value, all eigenvalues become distinct, which accounts for the result of a single driver node with *n*_m_=1/*N*. This relation holds as 〈*k*〉 is increased further.

We also find that random link weights have little effect on *n*_m_ for ER networks (e.g. comparing figure 2*a* with figure 2*b*), due to the fact that an ER network tends to have many isolated components. By contrast, for SF networks, random link weights can induce a dramatic difference from the case of identical link weights, as shown in figure 2*c* with figure 2*d*. Particularly, a single messenger node is sufficient to locate sources for random link weights with weak noise, regardless of the values of 〈*k*〉 and *N*. This phenomenon can be explained based on equation (2.5), where random link weights can be regarded as imposing perturbation to the eigenvalues of the relevant unweighted Laplacian matrix (the locations of non-zero elements in the two matrices are the same). If the network has a single component, the unweighted Laplacian matrix has only one zero eigenvalue in the spectrum. The random link weights will shift the non-zero eigenvalues in the spectrum, making the probability of finding two or more identical eigenvalues effectively zero. We then expect to find one null eigenvalue and *N*−1 distinct non-zero eigenvalues so that the entire spectrum contains eigenvalues that are all distinct. As a result, according to equation (2.5), we have *N*_m_=1 for the undirected, single-component SF network with random link weights. A generalization is that, for an arbitrary undirected network with random link weights and multiple components, the value of *N*_m_ is exclusively determined by the number of components, *N*_c_, i.e. *N*_m_=*N*_c_, due to the fact that each component contributes a null eigenvalue. Consequently, the maximum eigenvalue degeneracy that determines *N*_m_ is equal to the number of components, *N*_c_.

We now turn to directed ER and SF networks. For unidirectional links in such a network, the average degree of the network is 〈*k*〉=〈*k*_out_〉/2=〈*k*_in_〉/2, where *k*_out_ and *k*_in_ denote the out-degree and in-degree, respectively. For directed ER networks, the FE formula is equation (2.6) with *a*=0. Analytical prediction of *n*_m_ can be obtained based on the FE (see electronic supplementary material, S4)

2.8 $$n_{m}^{\mathrm{DER}}\approx e^{-\langle k\rangle }+\frac{{\left\langle k\right\rangle }^{2}\;e^{-2\langle k\rangle }}{4}.$$

For directed SF networks, the FE formula is still equation (2.6) with *a*=0, −1 or −2 (see electronic supplementary material, S4). The quantity *n*_m_ can be theoretically predicted via (see electronic supplementary material, S4)

2.9 $$n_{m}^{\mathrm{DSF}}\approx \sum_{k=1}^{N-1}2^{-k}P(k),$$

where *k* is node degree and *P*(*k*)=*P*(*k*_in_+*k*_out_) is the degree distribution. Figure 4*e*–*h* shows, for directed ER and SF networks, the results of *n*_m_ from FE and analytical prediction agree well with those from ET without any approximations.

It is noteworthy that for directed networks with random link weights, *N*_m_ is not determined by the number of components, *N*_c_, because there can be more than one zero in the eigenvalue spectrum of a component, a situation that differs from that for undirected networks. In particular, for a directed network, the matrix *L* can have any number of zero diagonal elements because any node without outgoing links corresponds to such a diagonal element. According to the minimum output analysis, there can then be any number of messenger nodes in a component. As a result, in contrast with undirected networks with random weights, the quantity *N*_m_ in directed networks with random link weights should be calculated by using either equation (2.4) or equation (2.6) for sparse networks, not by counting the number of disconnected components.

### 2.4. Source locatability of real networks

**Figure 3.**
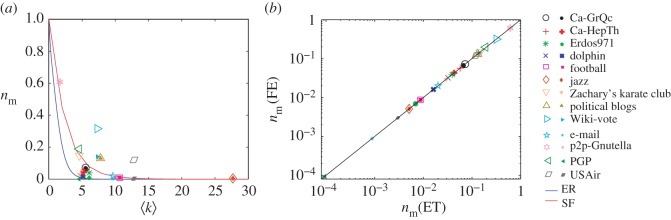
Source locatability of empirical networks. (*a*) The locatability measure *n*_m_ as a function of average degree 〈*k*〉 for a number of real social and technological networks, on which diffusion and spreading processes may occur. (*b*) The locatability measure obtained by using exact theory *n*_m_(ET) (equation (2.4) or equation (2.5)) and obtained by using fast estimation *n*_m_(FE) (equation (2.6)) of real networks. Here, 〈*k*〉=〈*k*_in_〉/2=〈*k*_out_〉/2 for a directed network. Theoretical results of ER network (equation (2.7)) and SF network with *γ* = 3 (equation (2.9)) are shown as a reference. Hollow symbols represent the results of unweighted real networks and solid symbols represent the results of real networks with random link weights selected from a uniform distribution in the range (0,2). More details of the real networks can be found in electronic supplementary material, S6 and table S1.

We also investigate the source locatability *n*_m_ for a number of empirical social and technological networks, on which diffusion or spreading processes may occur. Because of the lack of link weights in the real networks, we consider two typical scenarios, unweighted networks and random weight distribution. As shown in figure 3*a*, *n*_m_ for an unweighted real network is always larger than or equal to that of the network with random weights, indicating that random link weights are beneficial to source localization. Another feature is that sources in the technological networks with heterogeneous degree distribution (e.g. Wiki-vote, p2p-Gnutella, PGP, Political blogs, USAir) are usually more difficult to be located than the social networks with relatively homogeneous degree distribution.

We also test the practical feasibility of our fast estimation approaches by using the real networks. As shown in figure 3*b*, we obtain a good agreement between *n*_m_(ET) based on the exact locatability theory with high computational complexity and *n*_m_(FE) from the fast estimation with much higher efficiency for both unweighted and weighted real networks with random weights. These results validate our fast estimation approach as applied to real networks. (The characteristics of the real networks are described in electronic supplementary material, S6 and table S1).

Combining the results of real and model networks, we discover that the average node degree, the degree distribution and the link weight distribution jointly determine the source locatability. In particular, sources in networks with a homogeneous degree distribution, more connections and random link weights are more readily to be located.

### 2.5. Identification of messenger node set

**Figure 4.**
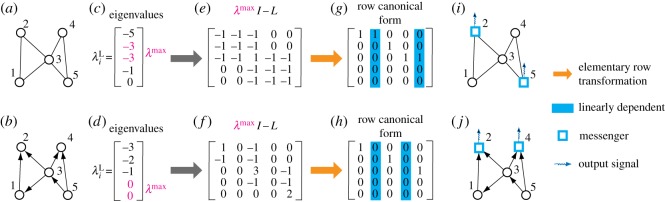
Identification of messengers. (*a*–*b*) Illustration of our method to identify messenger nodes for (*a*) a simple undirected network and (*b*) a simple directed network. (*c*–*d*) Eigenvalues of the undirected network in (*a*) and that of the directed network in (*b*), respectively. In (*c*) and (*d*), the eigenvalue $\lambda^{\max}$ corresponding to the maximum geometric multiplicity $\mu (\lambda^{\max})$ is highlighted in red. (*e*–*f*) Matrix $\lambda^{\max}I-L$ for the network in (*a*) and (*b*), respectively, where $\lambda^{\max}$ is highlighted. (*g*–*h*) Row canonical form of the matrix in (*e*) and (*f*) as a result of elementary row transformations, respectively. Here, linearly dependent columns in (*g*) and (*h*) are highlighted in blue. (*i*–*j*) Messenger nodes corresponding to the linearly dependent columns in the network in (*a*) and (*b*), respectively, and output signals produced by messenger nodes. For the network in (*a*) and (*b*), the configuration of messengers is not unique as it depends on the elementary row transformation, but the number of messengers *N*_m_ is fixed and solely determined by $\mu (\lambda^{\max})$.

We demonstrate how the *N*_m_ messenger nodes can be identified using the theory of exact observability of complex networks [19]. In particular, according to the classic PBH test theory [28] and our locatability theory, the output matrix *C* associated with the *N*_m_ messenger nodes satisfies the rank condition $\mathrm{rank}\left(\begin{array}{c} \lambda^{\max}I-L\\ C \end{array}\right)=N$, where $\lambda^{\max}$ is the eigenvalue with the maximum geometric multiplicity $\mu (\lambda^{\max})$ of matrix *L*, i.e. $N-\mathrm{rank}(\lambda^{\max}I-L)$ reaches maximum value that is nothing but *N*_m_ (see equation (2.4); electronic supplementary material, S3). Messenger nodes can be identified insofar as the output matrix *C* is determined. The computation complexity of our elementary transformation is $O(N^{2}{\left(\log N\right)}^{2})$ [37]. Figure 4*a*–*j* illustrates, for an undirected and a directed network, the working of our method of identifying the messengers. For each case, we first compute the eigenvalues $\lambda_{i}^{L}$ of the matrix *L* and find the eigenvalue $\lambda^{\max}$ corresponding to $\mu (\lambda^{\max})$. We then implement elementary row transformation on $\lambda^{\max}I-L$ to obtain its row canonical form that reveals a set of linearly dependent columns. The messenger nodes are nothing but the nodes corresponding to the columns that are linearly dependent on other columns. The minimum number of messenger nodes (linearly dependent columns) is exactly *N*_m_. Note that alternative configurations of the messenger nodes are possible. For example, as shown in figure 4*g*, we find that columns 1 and 2, and columns 4 and 5 are linearly correlated, requiring two messengers. As a result, there are four equivalent combinations for the messenger nodes: (1, 4), (1, 5), (2, 4) and (2, 5), any of which can be chosen.

## 3. Source localization based on compressive sensing

A result from the canonical observability theory is that, in order to fully reconstruct **x**(*t*_0_) from solutions of equation (2.3), at least *N*-step measurements from the messenger nodes are necessary. However, for our localization problem, the sources are ‘minority’ nodes in the sense that the number of sources is much smaller than the network size. In fact, the states of most nodes in the network are zero initially, indicating that the vector **x**(*t*_0_) is sparse with a large number of zero elements. The sparsity of **x**(*t*_0_) can be exploited to greatly reduce the measurement requirement. In particular, in the CS framework for sparse signal reconstruction [22,38], equation (2.3) can be solved and accurate reconstruction of **x**(*t*_0_) can be achieved through solutions of the following convex-optimization problem

3.1 $$\min∥\text{x}(t_{0}){∥}_{1}\;\mathrm{subject}\;\mathrm{to}\;\text{Y}=O\cdot \text{x}(t_{0}),$$

where $∥\text{x}(t_{0}){∥}_{1}=\sum_{i=1}^{N}|{\text{x}}_{i}(t_{0})|$ is the *L*_1_ norm of **x**(*t*_0_), $\text{Y}\in R^{qM}$, $O\in R^{qM\times N}$ and $\text{x}(t_{0})\in R^{N}$.

If *O* satisfies the restricted isometry property (RIP) [39], a full reconstruction of **x**(*t*_0_) can be guaranteed theoretically through *M*-step measurements via some standard optimization method, where *M* is much smaller than *N*. For realistic complex networks, the RIP may be violated, but because of the linear independence of rows in matrix *O* it is still feasible to reconstruct **x**(*t*_0_) from sparse data, where *M* can still be much smaller than *N*. Another advantage associated with the CS framework lies in its robustness against noise. Especially, to obtain the direct solution of **x**(*t*_0_) is not possible when there is measurement noise or measurements are not sufficient (*M*<*N*), but the CS framework overcomes these difficulties.

**Figure 5.**
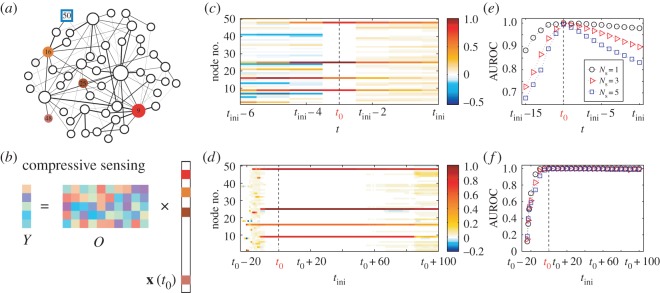
An example of locating sources in undirected weighted SF networks. (*a*) Illustration of an SF network with four sources with colours representing the initial state values. One messenger node is specified as a blue square. The thickness of the links represents their weight and the sizes of the nodes indicate their degrees. (*b*) The form of **Y**=*O***x**(*t*_0_) and the sparse initial state vector **x**(*t*_0_) to be reconstructed by using compressive sensing from a relatively small amount of data. (*c*) Reconstructed state *x*_*i*_(*t*) of each node for *t*≤*t*_ini_, where the initial observation time is *t*_ini_ (*t*_ini_≥*t*_0_). Colours represent the values of *x*_*i*_(*t*) with *t*≤*t*_ini_. (*d*) Reconstructed initial state *x*_*i*_(*t*_0_) of each node from different initial observation time *t*_ini_ when *t*_0_, the true triggering time, is being successfully inferred. Colours represent the reconstructed values of *x*_*i*_(*t*_0_). The colours have the same meanings as those in (*a*). The four sources are randomly selected and their *x*_*i*_(*t*_0_) values are larger than zero. (*e*) Area under a receiver operating characteristic (AUROC) as a function of *t* (*t*≤*t*_ini_) for a fixed initial observation time *t*_ini_. (*f*) AUROC versus *t* for different initial observation time *t*_ini_ and different number of sources (*N*_s_). Network parameters are set as follows. Network size is *N*=50, the average degree is 〈*k*〉=4, and the random link weights are selected from a uniform distribution in the range (0,2). For the diffusion dynamics, we set the diffusion parameter to be *β*=0.05 and the initial state of sources in **x**(*t*_0_) is randomly selected from a uniform distribution in the range (0.1,1). To implement the source localization process, the parameters are: noise amplitude *σ*=0, data=0.5, and the results are obtained by averaging over 300 independent simulations.

A complete description of our framework to reconstruct the initial states with unknown *t*_0_ is described in §5.2. Here, we present an example of locating diffusion sources in an SF network, as shown in figure 5. For an SF network of a single connected component and random link weights, our minimum output analysis gives *N*_m_=1, and the single messenger node can be selected arbitrarily. As shown in figure 5*a* for an SF network with four sources and a single messenger node. For convenience, we define data≡*M*/*N*, i.e. the ratio of the utilized amount of measurement to the amount required by the canonical observability theory. Figure 5*b* shows the form of **Y**=*O***x**(*t*_0_), in which the initial state vector **x**(*t*_0_) is to be reconstructed. Note that **x**(*t*_0_) is quite sparse with four non-zero elements corresponding to the four sources. Thus, **x**(*t*_0_) can be reconstructed by using the compressive sensing from a relatively small amount of data. Figure 5*c* shows, for data=0.5 and in the absence of noise, four sources and their locations as well as the initial (triggering) time *t*_0_ can be accurately inferred, even though *t*_0_ is unknown. We see that the reconstructed state **x**(*t*_ini_−3) is the sparsest in the sense that it is sparser than all the other states before and after *t*_ini_−3. This indicates that the initial time is *t*_0_=*t*_ini_−3 and **x**(*t*_ini_−3) is the initial state, in which *x*_*i*_(*t*_ini_−3) with non-zero values correspond to sources.

An alternative criterion for inferring initial time *t*_0_ is that **x**(*t*_0_) is non-negative but some elements in **x**(*t*_0_−1) are negative. The presence of negative values in **x**(*t*_0_−1) is because of the violation of physical process at time *t*_0_−1. Actually, the diffusion process at *t*_0_−1 does not exist, such that there is no physical solution of **x**(*t*_0_−1), regardless of using any methods to solve **x**(*t*_0_−1). A forced solution of **x**(*t*_0_−1) will account for unreasonable values in **x**(*t*_0_−1). As a result, negative values in **x**(*t*_0_−1), **x**(*t*_0_−2),… are highly possible, and offer an alternative way to the sparsity **x**(*t*) for inferring *t*_0_.

In this manner, not only can we locate the sources but we can also infer the initial states of the source nodes. As shown in figure 5*c*, the reconstructed initial state values of the sources at *t*=*t*_0_ are in good agreement with those shown in figure 5*a* (see §5.2 for more details). Figure 5*d* shows how different initial observation time *t*_ini_ affects source localization. We find that, in the wide range of *t*_ini_ from *t*_ini_=*t*_0_−10 to *t*_ini_=*t*_0_+80, four sources can be precisely located from a small amount of data. Here, *t*_ini_<*t*_0_ indicates that we started to observe messenger nodes prior to the occurrence of the diffusion event from the four sources, which is possible because *t*_0_ is unknown. If *t*_ini_ is much earlier than *t*_0_, the spreading process may not occur after *M*-step measurements, rendering source localization impossible using any method in principle. This accounts for the failure of our method for *t*_ini_<*t*_0_−20. Also, if *t*_ini_ is much later than *t*_0_, computing errors and noise effect will be amplified by using the CS-based optimization, leading to the inaccuracy of source localization, e.g. *t*_ini_>*t*_0_+90. These issues notwithstanding, our method is quite effective for a vast range of *t*_ini_ for multiple sources based on sparse data from a minimum number of messenger nodes.

To characterize the performance of our source localization method, we use a standard index from signal processing, the area under a receiver operating characteristic (AUROC) [40,41]. In particular, AUROC=1 indicates the existence of a threshold that can entirely separate the initial states **x**(*t*_0_) of the sources from other nodes in the network, giving rise to perfect localization of sources (see electronic supplementary material, S7 for the detailed definition of AUROC). To give a concrete example, we set *t*_ini_=*t*_0_+10. Figure 5*e* shows that the value of AUROC reaches unity at *t*_ini_−10, namely *t*_0_, demonstrating a nearly perfect localization of sources with different number. The highest reconstruction accuracy at *t*=*t*_0_ corresponds to the highest sparsity of the reconstructed state at *t*_0_ in figure 5*c*. For *t*>*t*_0_, at an arbitrary time *t*′, the number of nodes with non-zero states will be larger than the number of sources, because of the diffusion from sources to the other nodes. Thus, one may not distinguish sources from the other nodes based on the reconstructed **x**(*t*′), accounting for the lower values of AUROC at *t*′ compared with that at *t*_0_. On the other hand, consider an arbitrary time *t*′′ with *t*′′<*t*_0_. At *t*′′, the spreading process has not occurred, and there is no causality between the states at *t*′′ and the observation. When we impose the reconstruction on **x**(*t*′′), we cannot obtain the true **x**(*t*′′) with all zero elements but a virtual initial state vector with certain errors when compared with **x**(*t*_0_). The reconstruction errors will cause more non-zero states on the basis of **x**(*t*_0_), inducing a denser state vector than **x**(*t*_0_) and therefore lower values of AUROC. The reconstruction errors also explain the fact that the value of AUROC decreases more rapidly for *t*<*t*_0_ than for *t*>*t*_0_. Figure 5*f* shows the statistical results of figure 5*d*. We see that AUROC reaches unity when the observation time *t*_ini_ is about 3 time steps ahead of *t*_0_, and the AUROC value is nearly unchanged as *t*_ini_ is further increased, which is consistent with the phenomena shown in figure 5*d*. (In addition, examples of locating sources in ER networks with and without measurement noise, and in SF networks with measurement noise are presented in electronic supplementary material, S8 and figures S1–S3.) Here, we choose the node number 50, i.e. no. 50, to be the messenger. We also find the different choices of messengers do not affect the result of the sources localization, see electronic supplementary material, S8 and figure S4 for the details. We also investigate effects of the network size on the sources localization, and find that the data will be smaller for a larger network size when AUROC reaches 1, see electronic supplementary material, S8 and figure S5. This is because that the initial state **x**(*t*_0_) is sparser when the network size is larger, for a certain AUROC, then the amount of data will be smaller by using CS methods.

**Figure 6.**
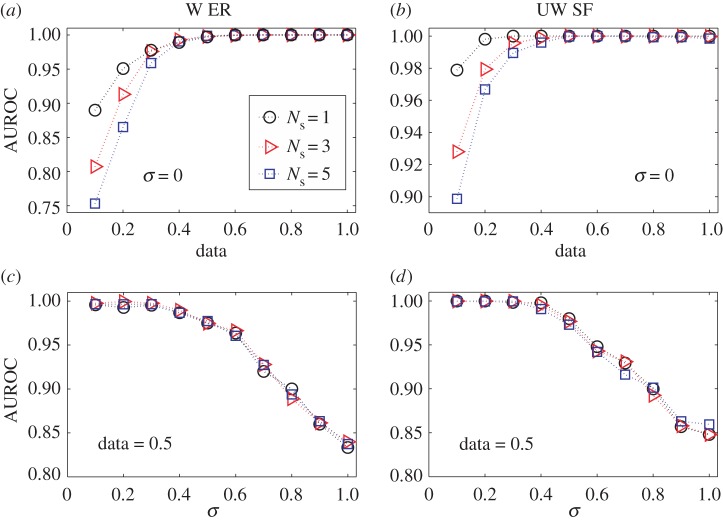
Locatability performance in undirected ER and SF networks. (*a*–*d*) AUROC as a function of data for (*a*) weighted ER and (*b*) unweighted SF networks, and as a function of noise variance *σ* for (*c*) weighted ER and (*d*) unweighted SF networks. In (*a*) and (*b*), *σ* is fixed at 0. In (*c*) and (*d*), data are fixed at 0.5. Cases with different numbers of sources, *N*_s_, are included. For a random guess, the AUROC value is 0.5. The average degree 〈*k*〉 is 2 and 4 for the ER and SF networks, respectively. We set *β*=0.1 for ER networks and *β*=0.05 for SF networks. The results are obtained by averaging over 500 independent simulations. The other parameters are the same as in figure 5.

We also systematically test the performance of our locatability framework with respect to data requirement and robustness against noise. We assume that measurements are contaminated by white Gaussian noise: $\hat{\text{y}}(t)=\text{y}(t)[I+N(\text{0},\sigma^{2}I)]$, where $\text{0}\in R^{N}$ is zero vector and $I\in R^{N\times N}$ is the identity matrix, and *σ* is the standard deviation. The results of AUROC as a function of data for ER and SF networks are shown in figures 6*a* and 6*b*, respectively. In the absence of noise (*σ*=0), even for data=0.1, high values of AUROC can be achieved, e.g. 0.9, especially for SF networks. The value of AUROC exceeds 0.95 when the amount of data is 0.3, and reaches unity for data≥0.5. The essential feature holds in the presence of noise and for arbitrary values of *N*_s_ (see electronic supplementary material, S9 and figure S6). Another finding is that, fewer sources (smaller values of *N*_s_) require less data, due to the fact that a sparser **x**(*t*_0_) is induced as a result of smaller *N*_s_ and in general, the CS framework requires less data to reconstruct a sparser vector. Systematic results on noise resistance are shown in figures 6*c*–*d*, where we see that the AUROC value is nearly indistinguishable across different numbers of sources, *N*_s_. This is different from the results in figure 6*a,b*, and there is almost no difference between the results from ER and SF networks. Figure 6*c,d* also shows that, as *σ* is increased from 0 to 1, the AUROC value is only slightly reduced (AUROC≈0.85 for *σ*=1), indicating the extraordinary robustness of our locatability framework against noise. We also study the effect of the diffusion parameter *β* on source localization with respect to different data amounts and values of the noise variance. We find that *β* has little influence on the accuracy of source localization (see electronic supplementary material, S10 and figures S7–S9).

## 4. Discussion

We developed a framework for locating sources of diffusion or spreading dynamics in arbitrary complex networks (directed or undirected, weighted or unweighted) based solely on sparse measurement from a minimum number of messenger nodes. The key to the general framework lies in combining the controllability theory of complex networks with the compressive sensing paradigm for sparse signal reconstruction, both being active areas of research in network science and engineering. Particularly, the minimum set of messenger nodes can be identified efficiently using the minimum output analysis based on exact controllability of complex networks and the dual relation between controllability and observability. The ratio of the minimum messenger nodes to the network size characterizes the source locatability of complex networks. We find that sources in a denser and homogeneous network are more readily to be located, which distinguishes our work from those in the literature based on alternative algorithms. A finding is that, for undirected networks with one component, random link weights and weak noise, a single messenger node is sufficient to locate sources at any locations in the network. By using the data from the minimum set of messenger nodes, an approach based on compressive sensing is offered to precisely infer the initial time, at which the diffusion process starts, and the sources with non-zero states initially. Because the initial state vector to be recovered for source localization is generically sparse, compressive sensing can be employed to locate the sources from small amounts of measurement, making our framework robust against insufficient data and noise. Practically, the highlights of our framework consist of the following three features: minimum messenger nodes, sparse data requirement and strong noise resistance, which allow the sources of dynamical processes to be identified accurately and efficiently.

Our approach was partially inspired by the pioneering effort in connecting the conventional observability theory for canonical linear dynamical systems with the compressive sensing approach [42–44]. To our knowledge, the source locatability problem has not been tackled in such a comprehensive way prior to our work. The minimal output analysis based on the controllability and observability theory for complex networks deepens our understanding of the dynamical processes on complex networks, which finds applications, e.g. in the design and analysis of large-scale sensor networks. Incorporating compressive sensing to uncover the sources and the original time of diffusion represents an innovative approach to a practical problem of significant interest but limited by finite resources for collecting data and by measurement or background noise. The underlying principle of the framework can potentially be applied to solving other optimization problems in complex networks. While we study diffusion models on time-invariant complex networks, our general framework provides significant insights into the open problem of developing source localization methods for time-variant complex networks hosting nonlinear diffusion processes.

## 5. Methods

### 5.1. The main localization formula

The detailed form of **Y**=*O*⋅**x**(*t*_0_) is

5.1 $$\left(\begin{array}{c} \text{y}(t_{0})\\ \text{y}(t_{0}+1)\\ \vdots \\ \text{y}(t_{0}+N-1) \end{array}\right)=\left(\begin{array}{c} C\\ C[I+\beta L]\\ \vdots \\ C{\left[I+\beta L\right]}^{N-1} \end{array}\right)\text{x}(t_{0}),$$

where *N* time steps of measurements are necessary to ensure full rank of the observability matrix *O*. Insofar as *O* is of full rank, according to the canonical observability theory, there exists a unique solution of the initial states to the main localization function.

### 5.2. Reconstruction of initial state **x**(*t*_0_) without knowledge of initial time *t*_0_

For realistic diffusive processes on networks, the initial time *t*_0_ is usually not known *a priori*, making inference of the initial state **x**(*t*_0_) a challenging task. Taking advantage of the sparsity of the initial vector **x**(*t*_0_) and the underlying principle of compressive sensing, we articulate an effective method to uncover both **x**(*t*_0_) and *t*_0_ from limited measurements.

Say the initial observation time is *t*_ini_ (*t*_ini_≥*t*_0_). Considering all possible *t*_0_ ahead of *t*_ini_, we need to reconstruct a series of states, i.e. **x**(*t*_ini_), **x**(*t*_ini_−1), ⋯ , $\text{x}(t_{0}^{\prime })$ to ensure that the actual *t*_0_ lies in between *t*_ini_ and *t*_0_′. The series of states can be reconstructed from the uninterrupted observation **y**(*t*_ini_),…,**y**(*t*_ini_+*N*−1) according to the following equations:

5.2 $$\begin{array}{rl} \left(\begin{array}{c} \text{y}(t_{\mathrm{ini}})\\ \text{y}(t_{\mathrm{ini}}+1)\\ \vdots \\ \text{y}(t_{\mathrm{ini}}+N-1) \end{array}\right) & =\left(\begin{array}{c} C\\ C[I+\beta L]\\ \vdots \\ C{\left[I+\beta L\right]}^{N-1} \end{array}\right)\text{x}(t_{\mathrm{ini}}),\\ \left(\begin{array}{c} \text{y}(t_{\mathrm{ini}})\\ \text{y}(t_{\mathrm{ini}}+1)\\ \vdots \\ \text{y}(t_{\mathrm{ini}}+N-1) \end{array}\right) & =\left(\begin{array}{c} C[I+\beta L]\\ C{\left[I+\beta L\right]}^{2}\\ \vdots \\ C{\left[I+\beta L\right]}^{N} \end{array}\right)\text{x}(t_{\mathrm{ini}}-1)\\ & \vdots \\ \;\mathrm{and}\;\left(\begin{array}{c} \text{y}(t_{\mathrm{ini}})\\ \text{y}(t_{\mathrm{ini}}+1)\\ \vdots \\ \text{y}(t_{\mathrm{ini}}+N-1) \end{array}\right) & =\left(\begin{array}{c} C{\left[I+\beta L\right]}^{t_{\mathrm{ini}}-t_{0}^{\prime }}\\ C{\left[I+\beta L\right]}^{t_{\mathrm{ini}}-t_{0}^{\prime }+1}\\ \vdots \\ C{\left[I+\beta L\right]}^{t_{\mathrm{ini}}-t_{0}^{\prime }+N-1} \end{array}\right)\text{x}(t_{0}^{\prime }). \end{array}$$

The reconstruction process is terminated and *t*_0_ can be inferred if a sparsest state is identified, say **x**(*t*_1_), i.e. **x**(*t*_1_) is sparser than all reconstructed states at time before and after *t*_1_. Then, **x**(*t*_1_) is taken as the initial state with the initial time *t*_0_=*t*_1_.

By exploiting the natural sparsity of **x**(*t*), the CS framework for sparse signal reconstruction allows us to reconstruct **x**(*t*_ini_), $\text{x}(t_{\mathrm{ini}}-1),\ldots ,\text{x}(t_{0}^{\prime })$ iteratively from a small amount of data, i.e. *M*-step measurements and *M*<*N*, i.e. $\text{Y}\in R^{qM}$, $O\in R^{qM\times N}$ and $\text{x}(t_{0}^{\prime })\in R^{N}$. By contrast, at least *N*-step measurements are required in the conventional observability theory (equation (5.2)), where *M* depends on the sparsity of the state vector. In general, *M* can be much smaller than *N*, insofar as the number of sources *N*_s_ is much smaller than the network size *N*. According to equations (3.1) and (5.2), **x**(*t*_ini_), **x**(*t*_ini_−1), …, $\text{x}(t_{0}^{\prime })$ can be reconstructed efficiently from a small amount of observation that is much smaller than that required in the conventional observability theory.

## Supplementary Material

Supplementary materials for optimal localization of diffusion sources in complex networks

## Data accessibility

Data can be accessed at http://sss.bnu.edu.cn/%7Ewenxuw/data%5fset.htm.
