# Supplementary material for: Optimal localization of diffusion sources in complex networks
Source: R Soc Open Sci. 2017 Apr 12;4(4):170091. doi: 10.1098/rsos.170091 (PMC5414272; doi:10.1098/rsos.170091)
Supplement: Supplementary materials for optimal localization of diffusion sources in complex networks [file rsos170091supp1.pdf]

ROYAL SOCIETY OPEN SCIENCE

SUPPLEMENTARY MATERIALS for Optimal Localization of Diffusion Sources in Complex Networks

Zhao-Long Hu, Xiao Han, Ying-Cheng Lai, Wen-Xu Wang

S1. Constraint of diffusion parameter

In principle, to ensure that system (2.1) in the main text can characterize a diffusion process, the parameter β should be less than a certain critical value, such that state $x_i(t)$ can be confined in the range [0, 1] for an arbitrary node at any time t. Specifically, we rewrite system (2.1) in the main text as

$$\begin{aligned} x_i(t+1) &= x_i(t) - \beta \sum_{j=1, j\neq i}^{N} w_{ji} x_i(t) + \beta \sum_{j=1, j\neq i}^{N} w_{ij} x_j(t), \\ &= \left(1 - \beta \sum_{j=1, j\neq i}^{N} w_{ji}\right) x_i(t) + \beta \sum_{j=1, j\neq i}^{N} w_{ij} x_j(t). \end{aligned} \tag{S1}$$

To ensure that the state variable $x_i(t) \in [0, 1]$ for any node i, we must have

$$-\beta \sum_{j=1, j\neq i}^{N} w_{ij} x_j(t) \leq (1 - \beta \sum_{j=1, j\neq i}^{N} w_{ji}) x_i(t) \leq 1 - \beta \sum_{j=1, j\neq i}^{N} w_{ij} x_j(t), \tag{S2}$$

which leads to

$$\beta \leq \min_{i=1,2,\cdots,N} \frac{1}{\sum_{j=1, j\neq i}^{N} w_{ji}}. \tag{S3}$$

Thus, we set the diffusion parameter $\beta \in (0, \min_{i=1,2,\cdots,N} \frac{1}{\sum_{j=1, j\neq i}^{N} w_{ji}}]$, which can guarantee that the state $x_i(t)$ at any time is always subject to the range $[0, 1]$. As a result, system (2.1) is able to characterize real diffusion processes with $x_i(t)$ denoting the density of diffusion at node i. If the value of β exceeds the range, the state $x_i(t)$ may diverge.

S2. Locating sources in continuous-time dynamical networks

A linear, time-invariant, and continuous-time dynamical network system can be described in the following state-space form

$$\begin{cases} \dot{\mathbf{x}}(t) = A\mathbf{x}(t) \\ \mathbf{y}(t) = C\mathbf{x}(t), \end{cases} \tag{S4}$$

where $\mathbf{x}(t) \in \mathbb{R}^N$ represents the complete state of the network system at time t, N is the number of nodes, $\mathbf{y}$(t) is the vector of q outputs at time t, $A \in \mathbb{R}^{N\times N}$ is the system matrix, and $C \in \mathbb{R}^{q\times N}$ is the output matrix. If the full initial state of the system, $\mathbf{x}(t_0)$, can be obtained from the outputs in the time interval $[t_0,\ t]$, the system is observable [S1].

To be concrete, we present a general method of reconstructing the initial states of an arbitrary linear time-invariant network using the diffusion model

$$\dot{x}_i(t) = \beta \sum_{j=1}^{N} \left[w_{ij}x_j(t) - w_{ji}x_i(t) \right], \tag{S5}$$

where $x_i(t)$ is the state of node i at the time t, β is the diffusion coefficient (constant), and w_{ij} (w_{ji}) is the weight of a directed link from node j to i (i to j). Combining Eqs. (S5) and (S4), we have

$$\begin{cases} \dot{\mathbf{x}}(t) = \beta L\mathbf{x}(t) \\ \mathbf{y}(t) = C\mathbf{x}(t), \end{cases} \tag{S6}$$

where $L = (W - D)$, $W \in \mathbb{R}^{N\times N}$ is the adjacency matrix of elements w_{ij}, $D \in \mathbb{R}^{N\times N}$ is the diagonal matrix with element d_i representing the total out-weight $\sum_{j\in\Gamma_i} w_{ji}$ of node i (Γ_i is the set of neighbors of node i). The output response of the system is

$$\mathbf{y}(t) = Ce^{\beta L(t-t_0)}\mathbf{x}(t_0). \tag{S7}$$

For convenience, we can stack all the outputs $\mathbf{y}(t)$ into a vector: $\mathbf{Y} = [\mathbf{y}(t_0), \cdots, \mathbf{y}(t_0{+}0.1), \cdots, \mathbf{y}(t_0{+}0.2), \cdots, \mathbf{y}(t_0 + t)]^T$. Intuitively, N snapshot measurements of the network state are needed to achieve a unique solution. Without loss of generality, we sample the same time interval T to obtain

$$\begin{pmatrix} \mathbf{y}(t_0) \\ \mathbf{y}(t_0+T) \\ \vdots \\ \mathbf{y}(t_0+(N-1)T) \end{pmatrix} = \begin{pmatrix} C \\ Ce^{\beta LT} \\ \vdots \\ Ce^{(N-1)\beta LT} \end{pmatrix} \mathbf{x}(t_0) = O \cdot \mathbf{x}(t_0), \tag{S8}$$

where the matrix $O \in \mathbb{R}^{qN \times N}$ is the so-called observability matrix in canonical control theory. A unique solution of Eq. (S8) exists and the state vector $\mathbf{x}(t_0)$ at initial time is observable if and only if the rank condition $\mathrm{rank}(O) = N$ is satisfied [S2]. Our goal is to identify the minimum set of messenger nodes to satisfy the observability full rank condition.

To achieve our goal, we exploit the recently developed exact controllability theory [S1] and the duality between controllability and observability [S2], which enables us to find N_{m}, the minimum number of messengers in an efficient manner. In particular, for an arbitrary network, N_{m} is determined by the maximum geometric multiplicity $\max_i\{\mu(\lambda_i^L)\}$ of the eigenvalues λ_i^L of matrix L, as

$$N_{\mathrm{m}} = \max_i\{N - \mathrm{rank}[\lambda_i^L I - L]\}, \tag{S9}$$

which is exactly the same as that for discrete-time dynamical networks studied in the main text. Insofar as N_{m} is determined, the key to source localization is then to identify messengers to obtain the output matrix C. The method of identifying messengers is essentially the same as that for the discrete time case: by using the Popov-Belevitch-Hautus (PBH) test theory [S3], we obtain the output matrix C associated with N_{m} messenger nodes through $\mathrm{rank}\begin{pmatrix} \lambda^{\max} I - L \\ C \end{pmatrix} = N$, where $\lambda^{\max}$ is the eigenvalue corresponding to the maximum geometric multiplicity $\mu(\lambda^{\max})$ of matrix L. To determine the output matrix C, we implement elementary row transformation on matrix $\lambda^{\max} I - L$ to obtain the row canonical form of the matrix. The nodes whose numbers correspond to the linearly-dependent columns are the messenger nodes. Finally, combining with Eq. (S8), we can locate sources in continuous-time dynamic networks.

For continuous-time dynamics, by setting a small time interval T, we can recover state $\mathbf{x}(t)$ at any time interval t as that of the discrete-time dynamics in section 5.2 in the main text. Specifically, let t_{ini}

denote the initial observation time. Based on Eq. (S8), we have

$$\begin{aligned}
\begin{pmatrix} \mathbf{y}(t_{\text{ini}}) \\ \mathbf{y}(t_{\text{ini}}+T) \\ \vdots \\ \mathbf{y}(t_{\text{ini}}+(N-1)T) \end{pmatrix} &= \begin{pmatrix} C \\ Ce^{\beta LT} \\ \vdots \\ Ce^{(N-1)\beta LT} \end{pmatrix} \mathbf{x}(t_{\text{ini}}), \\
\begin{pmatrix} \mathbf{y}(t_{\text{ini}}) \\ \mathbf{y}(t_{\text{ini}}+T) \\ \vdots \\ \mathbf{y}(t_{\text{ini}}+(N-1)T) \end{pmatrix} &= \begin{pmatrix} Ce^{\beta LT} \\ Ce^{2\beta LT} \\ \vdots \\ Ce^{N\beta LT} \end{pmatrix} \mathbf{x}(t_{\text{ini}}-T), \\
&\vdots \\
\begin{pmatrix} \mathbf{y}(t_{\text{ini}}) \\ \mathbf{y}(t_{\text{ini}}+T) \\ \vdots \\ \mathbf{y}(t_{\text{ini}}+(N-1)T) \end{pmatrix} &= \begin{pmatrix} Ce^{(t_{\text{ini}}-t_0^{'})\beta LT} \\ Ce^{(1+t_{\text{ini}}-t_0^{'})\beta LT} \\ \vdots \\ Ce^{(N-1+t_{\text{ini}}-t_0^{'})\beta LT} \end{pmatrix} \mathbf{x}(t_0^{'}).
\end{aligned} \tag{S10}$$

We need to reconstruct a series of states, i.e., $\mathbf{x}(t_{\text{ini}})$, $\mathbf{x}(t_{\text{ini}}-T)$, $\cdots$, $\mathbf{x}(t_0^{'})$ to ensure that the actual t_0 lies in between t_{ini} and t_0'. Subsequently, we choose the sparsest state, say $\mathbf{x}(t_1)$, which is identified as the initial state and t_1 is the initial time t_0. However, it is not able to infer the exact t_0 in continuous-time dynamics. Instead, a very narrow range $t_1 \in [t_0-\epsilon, t_0+\epsilon]$ can be inferred, where ϵ is a very small positive number.

Therefore, our theoretical framework of source localization, including the minimum output theory for determining a minimum set of messenger nodes and identifying the messenger nodes is exactly the same for both discrete and continuous dynamical network systems.

S3. Proof of the minimum output theory

According to the exact controllability theory [S4] and the dual relation between network controllability and observability [S1], for system (2.2) in the main text, N_{m} is determined by the maximum geometric multiplicity of the eigenvalue λ_i of the matrix $I+\beta L$, i.e.,

$$N_{\text{m}} = \max_i \{N - \text{rank}[\lambda_i I - (I+\beta L)]\}. \tag{S11}$$

We can prove that $N_{\rm m}$ is independent of β in the sense that β can be eliminated from Eq. (S11). For matrix $I+\beta L$, we have

$$\begin{aligned}(I+\beta L)\mathbf{v} &= \mathbf{v}+\beta L\mathbf{v}, \\ &= \mathbf{v}+\beta\lambda_i^L\mathbf{v}, \\ &= (1+\beta\lambda_i^L)\mathbf{v},\end{aligned} \tag{S12}$$

where λ_i^L is the eigenvalue of matrix L and $\mathbf{v}$ is the associated eigenvector. Equation (S12) gives the eigenvalue λ_i of matrix $I+\beta L$, i.e.,

$$\lambda_i = 1+\beta\lambda_i^L. \tag{S13}$$

Inserting the expression of λ_i into Eq. (S11), we have

$$\begin{aligned}N_{\rm m} &= \max_i\{N-\text{rank}[(1+\beta\lambda_i^L)I-(I+\beta L)]\}, \\ &= \max_i\{N-\text{rank}[\beta(\lambda_i^L I-L)]\}, \\ &= \max_i\{N-\text{rank}[\lambda_i^L I-L]\},\end{aligned} \tag{S14}$$

which indicates that the minimum output $N_{\rm m}$ is independent of the value of β. The exact minimum output theory for arbitrary network [equation (2.4) in the main text] is proved.

For system (2.2) in the main text with an arbitrary undirected network, according to the exact controllability theory [S4] and the dual relation between network controllability and observability, $N_{\rm m}$ is determined by the maximum eigenvalue degeneracy of matrix $I+\beta L$, i.e.,

$$N_{\rm m} = \max_i\{\delta(\lambda_i)\}, \tag{S15}$$

where $\delta(\lambda_i)$ is the eigenvalue degeneracy of matrix $I+\beta L$. Equation (S13) demonstrates that there is a one-to-one correspondence between the eigenvalue λ_i of matrix $I+\beta L$ and the eigenvalue λ_i^L of matrix L. Thus, $I+\beta L$ and L have exactly the same eigenvalue degeneracy, which yields the exact minimum output theory [equation (2.5) in the main text] for undirected networks, i.e.,

$$N_{\rm m}^{\rm undirect} = \max_i\{\delta(\lambda_i^L)\}, \tag{S16}$$

and the diffusion parameter β is eliminated.

Furthermore, Eq. (S14) indicates that the geometric multiplicity of the eigenvalues of L is equal to that of $I+\beta L$, and the output matrix C of L is identical to that of $I+\beta L$ as well. Utilizing the

Popov-Belevitch-Hautus (PBH) test theory [S3], we can get the output matrix C from

$$\text{rank}\begin{pmatrix}\lambda^{\max}I - L\\ C\end{pmatrix} = N, \tag{S17}$$

instead of

$$\text{rank}\begin{pmatrix}\hat{\lambda}^{\max}I - (I+\beta L)\\ C\end{pmatrix} = N, \tag{S18}$$

where $\hat{\lambda}^{\max}$ is the maximum geometric multiplicity of the eigenvalues of $I + \beta L$. This implies that the diffusion parameter β has no influence on identifying messenger nodes based on PBH test as well, as described in the subsection *Identification of messenger node set*.

S4. Analytical treatment of locatability of ER and SF networks

In general, for an undirected network (symmetric matrix), eigenvalue degeneracy is exactly the same as its geometric multiplicity. Thus, according to the ET formulas, e.g., equations (2.4-2.5) in the main text, the eigenvalue $\lambda^{\max}$ with the maximum geometric multiplicity in equation (2.4) is nothing but the eigenvalue with the maximum degeneracy (the number of appearances in the eigenvalue spectrum). In this regard, if we are able to evaluate the eigenvalue with the maximum degeneracy a priori, the calculation of the all eigenvalues in equation (2.5) and that of matrix ranks for all possible eigenvalues in equation (2.4) can be saved, leading to a fast estimation of n_{m} by inserting the estimated eigenvalue into equation (2.4).

For a sparse undirected network (symmetric matrix), the diagonal elements of the network matrix often dominate eigenvalue spectrum [S5]. Hence, the diagonal elements with the maximum multiplicity (the largest number of appearances in the diagonal) could be a proxy of the eigenvalue with maximum degeneracy. However, for Laplacian matrix L, in additional to the diagonal elements, zero could dominate eigenvalue spectrum as well in the absence of any zero diagonal elements, because of the existence of isolated components. Note that each isolated component or node will contribute one null eigenvalue to the eigenvalue spectrum of a Laplacian matrix. Thus, diagonal elements and zero are possible candidates for formulating a fast estimation of the source locatability measure n_{m}:

$$n_{\text{m}}^{\text{sparse}} \approx 1 - \frac{\text{rank}(aI - L)}{N}, \tag{S19}$$

where a is either zero or the diagonal element of L with the maximum multiplicity. For the random

networks, the efficient formula (S19) can be further simplified. In particular, for small values of $\langle k \rangle$, due to the isolated nodes and the disconnected components, zero dominates the eigenvalue spectrum of the matrix L [S5] where, for example, each disconnected component generates at least one zero eigenvalue in L. For large values of $\langle k \rangle$, we expect all eigenvalues to be distinct without any dominant one. In this case, we can still choose zero to be the eigenvalue associated with a in Eq. (S19). Taken together, in a wide range of $\langle k \rangle$ values, the efficient formula Eq. (S19) holds with $a = 0$,

$$n_{\mathrm{m}}^{\mathrm{sparse}} \approx 1 - \frac{\mathrm{rank}(L)}{N}. \tag{S20}$$

In an undirected ER network with small connection probability, there are a number of isolated components, accounting for the dominance of zero in the eigenvalue spectrum. Thus, the source locatability n_{m} can be estimated by examining the isolated components. Let's define E as the number of edges in the network, and the average degree $\langle k \rangle = 2E/N$. If $\langle k \rangle \in [0, 1)$, then the average number of isolated components $N_{\mathrm{cc}} = N - E + O(1)$, where the bound of $O(1)$ depends only on $\langle k \rangle$ [S6]. Thus, the average ratio of isolated components n_{cc} is

$$n_{\mathrm{cc}} = 1 - (\langle k \rangle)/2 + O(1/N), \tag{S21}$$

If $\langle k \rangle \sim 1$, then $N_{\mathrm{cc}} = N - E + O(\log(N))$ [S6]. Similarly, in this case,

$$n_{\mathrm{cc}} = 1 - (\langle k \rangle)/2 + O(\log(N)/N), \tag{S22}$$

If $\langle k \rangle > 1$, we have

$$\lim_{N \to +\infty} n_{\mathrm{cc}} = \frac{1}{\langle k \rangle}(f(\langle k \rangle) - \frac{f(\langle k \rangle)^2}{2}), \tag{S23}$$

where $f = f(\langle k \rangle)$ is the only solution satisfying $f \in (0, 1)$ of the equation $fe^{-f} = 2fe^{-2f}$, namely, $f(\langle k \rangle) = \sum_{k=1}^{\infty} \frac{k^{k-1}}{k!}(\langle k \rangle e^{-\langle k \rangle})^k$.

Based on the above three equations, in thermodynamic limit, we have

$$n_{\mathrm{m}}^{\mathrm{UER}} = \begin{cases} 1 - \langle k \rangle/2 & \langle k \rangle \in [0, 1] \\ \frac{1}{\langle k \rangle}(f(\langle k \rangle) - f(\langle k \rangle)^2/2) & \langle k \rangle \in (1, \infty). \end{cases} \tag{S24}$$

For a directed network, let k_{out} and k_{in} be the out-degree and in-degree, respectively, and suppose that the links are unidirectional. The average degree of the network is $\langle k \rangle = \langle k_{\mathrm{out}} \rangle/2 = \langle k_{\mathrm{in}} \rangle/2$. A fast estimation of the source locatability yields

$$n_{\mathrm{m}}^{\mathrm{sparse}} \approx 1 - \frac{\mathrm{rank}(aI - L)}{N}, \tag{S25}$$

where a is 0, -1 or -2, due to the fact that the diagonal element of matrix L is dominated by 0, -1 or -2 for small average degree $\langle k\rangle$. Numerical calculations suggest that the main contributions to $n_{\rm m}$ come from eigenvalues 0, -1 and -2.

For a directed ER network with a small connection probability $2\langle k\rangle/N$, analogous to the undirected case, we only need to consider isolated nodes and nodal pairs to obtain

$$n_{\rm m}^{\rm DER} \simeq \max\{1/N,\ P(k_{\rm out}=0,k_{\rm in}=0)+P(k_{\rm out}=1,k_{\rm in}=0)P(k_{\rm out}=0,k_{\rm in}=1)\}. \tag{S26}$$

Since $k_{\rm in}$ is independent of $k_{\rm out}$, we have

$$n_{\rm m}^{\rm DER} \approx e^{-\langle k\rangle}+\frac{\langle k\rangle^2 e^{-2\langle k\rangle}}{4}. \tag{S27}$$

For a directed SF network, nodes of zero out-degree must be the messengers. In this case, we can estimate $n_{\rm m}$ as

$$n_{\rm m}^{\rm DSF} \simeq \max\{1/N,\ \sum_{k=1}^{N-1} P(k_{\rm out}=0|k)P(k)\}, \tag{S28}$$

where $P(k)=P(k_{\rm in}+k_{\rm out})$ follows a power law, and $P(k_{\rm out}|k)$ is the conditional probability that one node has out-degree $k_{\rm out}$ when its degree is k. According to binomial theorem, we have

$$P(k_{\rm out}|k)=\binom{k}{k_{\rm out}}\left(\frac{1}{2}\right)^{k_{\rm out}}\left(\frac{1}{2}\right)^{k-k_{\rm out}}, \tag{S29}$$

which yields

$$P(k_{\rm out}=0|k)=2^{-k} \tag{S30}$$

and consequently,

$$n_{\rm m}^{\rm DSF} \approx \sum_{k=1}^{N-1} 2^{-k}P(k). \tag{S31}$$

S5. Cavity method for estimating locatability

In Ref. [S7], the cavity method was used to quantify the network controllability via the density of the driver nodes for directed networks. The method was subsequently extended to undirected networks [S4]. Because of the duality between controllability and observability, we can use the cavity method to estimate the locatability $n_{\rm m}$ for general complex networks. In particular, for a directed network with similar in-

and out-degree distribution $P(k)$, the locatability n_m is given by

$$n_\mathrm{m} = G(w_2) + G(1 - w_1) - 1 + \langle k \rangle w_1 (1 - w_2), \tag{S32}$$

where $G(x)$ is the generating function satisfying

$$G(x) = \sum_{k=0}^{\infty} P(k) x^k. \tag{S33}$$

The quantities w_1 and w_2 in Eq. (S32) can be obtained through the following self-consistent equations:

$$w_1 = H[1 - H(1 - w_1)], \tag{S34}$$

$$w_2 = 1 - H[1 - H(w_2)], \tag{S35}$$

where $H(x)$ is a generating function defined as

$$H(x) = \sum_{k=0}^{\infty} Q(k+1) x^k, \tag{S36}$$

and $Q(k) = kP(k)/\langle k \rangle$.

In Ref. [S5], the cavity method was used to calculate the controllability measure for directed networks with multiple types of self loops, where the diagonal elements of matrix L were regarded as self loops. This is key to calculating the locatability measure n_m for matrix $L - aI$. Adopting the method in Ref. [S5], we have that, if a diagonal element of $L - aI$ is nonzero, the in- and out-degree of the corresponding node is increased by 1, while the degrees of the other nodes remain the same in the original weighted network. For an undirected network, if a diagonal element of $L - aI$ is nonzero, the degree of the node is increased by 2. Finally, based on the new node degrees, we can obtain n_m by combining Eqs. (S32-S36).

S6. Characteristics of real networks studied

In the main text, results from a number of real networks are presented to test our ET and FE methods. The details of the real networks are listed in table S1, which includes the names of the data sets, the data type, the number of nodes N, the number of links E, and brief descriptions of the networks.

S7. Performance assessment of source localization

The area under a receiver operating characteristic (AUROC) for the source localization is defined in terms of true positive rate (TPR) and false positive rate (FPR). TPR and FPR are defined as follows:

$$\mathrm{TPR}(s) = \frac{\mathrm{TP}(s)}{P} \quad \text{and} \quad \mathrm{FPR}(s) = \frac{\mathrm{FP}(s)}{Q}, \tag{S37}$$

where s is the cutoff (threshold) in the list of reconstructed state $x_i(t)$ at time t, $\mathrm{TPR}(s)$ ($\mathrm{FPR}(s)$) is the number of true (false) positives in the top s reconstructed values of $x_i(t)$ and P (Q) is the number of positives (negatives) in the gold standard. AUROC is the area under the TPR-FPR curve.

S8. More examples of source localization

1. An example of locating sources in undirected weighted ER network without noise

To be concrete, we set $\mathrm{data} = 0.5$ and assume that the initial triggering time t_0 is unknown. In figure S1(a), we show that measurements from any single node are sufficient to locate the sources, as the network has a single connected component with random link weights. Figure S1(b) shows that our method can accurately infer both the number of sources and their locations, as well as the initial triggering time t_0. Figure S1(c) shows, for different initial observation time t_{ini}, that the number of sources and their locations can be determined in a large range after t_0 is detected as in figure S1(b).

The performance of our method can be assessed, as follows. For concreteness, we assume $t_{\mathrm{ini}} = t_0 + 10$. Figure S1(d) shows that the value of AUROC will reach unity at $t_{\mathrm{ini}} - 10$, which is the triggering time t_0, and the AUROC value decreases more significantly when the inferred time is $t < t_0$ as compared with the case of $t > t_0$. From figure S1(e), we see that AUROC reaches unity when the observation time is $t_{\mathrm{ini}} \approx 3$ time steps before t_0, and AUROC is almost unchanged as t_{ini} is increased. These results are similar to those of SF networks (figure 5 in the main text).

2. Examples of locating sources in undirected weighted SF and ER networks in presence of noise

We extend our source localization framework to cases where there is noise for SF and ER networks. Assuming $\sigma = 0.5$ and setting $\mathrm{data} = 0.5$, we reconstruct $\mathbf{x}(t_0)$ with four random sources. Figure S2 and figure S3 show essentially the same results as figure S1, indicating the robustness of our localization framework.

3. Effect of different choices of messengers and network size N on source localization

Note that there may exist different choices of messenger nodes that are able to ensure $\mathrm{rank}\begin{pmatrix} \lambda^{\max} I - L \\ C \end{pmatrix} = N$, where $\lambda^{\max}$ is the eigenvalue with the maximum geometric multiplicity $\mu(\lambda^{\max})$ of matrix L. More details can be seen in section 2.5 in the main text. Here we explore the effect of different choices of messengers on sources localization and inferring the initial time t_0. In random weighted SF networks, any single messenger node can guarantee that the network is observable. Without loss of generality, we choose a node, e.g., number 10, 20, 30, 40 and 50 to be the messenger, respectively. Figure S4 shows that different choices of messengers produce similar accuracy in detecting diffusion sources and initial time t_0.

We also study the relationship between the required amount of data for accurate localization, data (M/N), and network size N. As shown in figure S5, to ensure full localization of diffusion sources (AUROC=1), for both ER and SF networks, data is about 0.4, 0.3 and 0.2 for N=50, 100 and 200, respectively. These results demonstrate that for the same number of sources, a network with larger size requires a relatively less amount of data. This phenomenon is because of the inherent property of CS approach, i.e., to reconstruct a sparser signal, less data are needed. Note that a fixed number of sources in a larger network are sparser than that in a smaller network. Thus, according to the property of CS approach, in general a relatively less amount of data can ensure full localization in a larger network.

S9. Robustness of our source localization framework

We systematically investigate source localization for undirected and weighted ER and SF networks in terms of data requirement and noise resistance. Supplementary figure S6(a) and figure S6(b) show, for $\sigma = 0$, relatively small data amount, and both ER and SF networks, that the value of AUROC increases when the number of sources is decreased. This means that the localization accuracy tends to increase with the sparsity of vector $\mathbf{x}(t_0)$. We also see that the value of AUROC exceeds 0.9 even though data amount is only 0.3, and unity value of AUROC can be achieved for data ≥ 0.5. For $\sigma = 0.5$, as shown in Supplementary figure S6(c) and figure S6(d), similar results are obtained, except that the corresponding AUROC values are slightly smaller. As can be seen from Supplementary figure S6(e) and figure S6(f), for data $= 0.5$, the AUROC values are nearly indistinguishable for different values of N_{s}. For $\sigma \leq 0.3$, the value of AUROC reaches unity but will decrease with σ for $\sigma > 0.3$, regardless of the values of N_{s}. The AUROC reaches unity with error less than 5% despite that σ is as large as 0.5. All the results provide additional evidence for the robustness of our method against noise and insufficient data.

S10. Effects of diffusion parameter

We investigate the effect of the diffusion parameter β on the accuracy of actual localization of sources based on compressed sensing approach. Firstly, we examine AUROC as a function of t ($t \leq t_{\text{ini}}$) for different values of β, as shown in figure S7. Indeed, we see that the value of β affects AUROC and smaller values of β results in higher values of AUROC. A heuristic explanation can be provided with respect to the relation between diffusion velocity and time scale. Note that larger values of β accounts for faster diffusion, whereas smaller values of β corresponds to slower diffusion. Thus, the effect of β is analogous to changing the time scale of the diffusion process. A smaller (larger) value of β corresponds to a larger (smaller) time scale. As shown in figure S7, for both ER and SF networks, if we modify the time scale, i.e., shrink the horizontal coordinate t for smaller values of β, and/or enhance the horizontal coordinate t for larger values of β, AUROC as a function of t associated with distinct values of β will be quite similar. Despite the effect of β, we note that the highest AUROC always occurs at $t = t_0$, regardless of the value of β. Based on the fact that the highest AUROC is at $t = t_0$, t_0 can be precisely inferred as well, regardless of the value of β. Furthermore, we find that, as shown in figures S8 and S9, the choice of different values of β has little influence on the accuracy of source localization at t_0 with respect to different data amounts and values of noise variance. We see that, as β is decreased, e.g., $\beta = 0.01$, the localization accuracy is slightly reduced (figure S8), due to the computational errors associated with the iterative process in the implementation of the compressive sensing algorithm.

Supplementary Figures

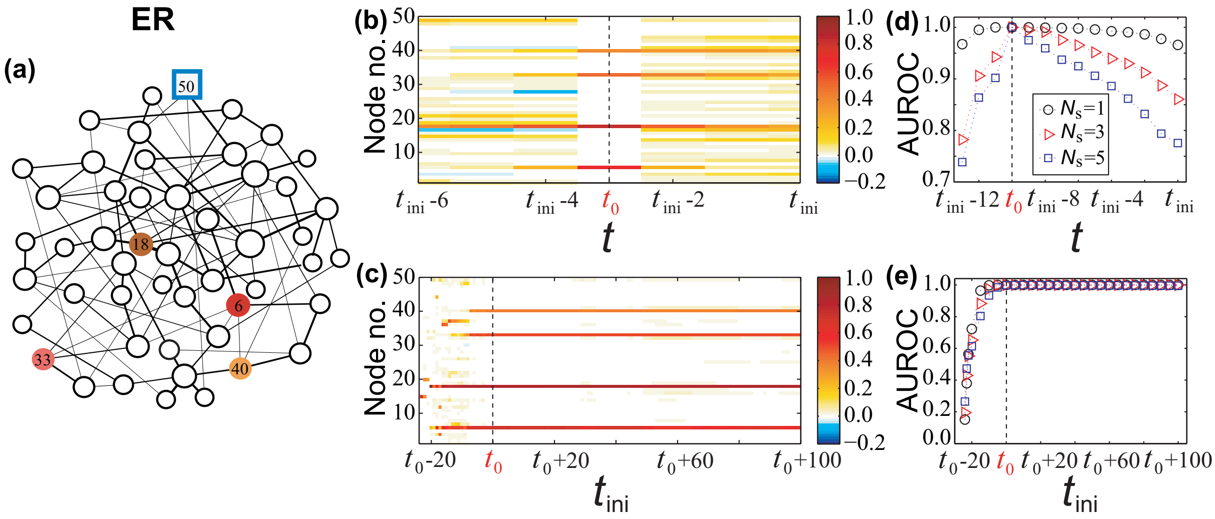

Figure S1. An example of locating sources in undirected weighted ER networks. (a) Illustration of a ER network with four sources with colors representing the initial state values. One messenger node is specified as a blue square. The thickness of links represents their weight and the size of the nodes corresponds to their degrees. (b) Reconstructed state $x_i(t)$ of each node for $t \leq t_{\text{ini}}$, where the initial observation time is t_{ini} ($t_{\text{ini}} \geq t_0$). Colors represent the values of $x_i(t)$ with $t \leq t_{\text{ini}}$. (c) Reconstructed initial state $x_i(t_0)$ of each node from different initial observation time t_{ini} when t_0, the true triggering time, is being successfully inferred. Colors represent the reconstructed values of $x_i(t_0)$. The colors have the same meanings as those in (a). The four sources are randomly selected and their $x_i(t_0)$ values are larger than zero. (d) AUROC as a function of t ($t \leq t_{\text{ini}}$) for a fixed initial observation time t_{ini}. (e) AUROC versus t for different initial observation time t_{ini} and different number of sources (N_s). For both (d) and (e), there is no noise, cases for different number of sources are illustrated, N_s, and t_0 is the true triggering time. In all cases, the network size is $N = 50$, the average degree is $\langle k \rangle = 4$, the link weights are uniformly distributed in $(0, 2)$, the diffusion parameter $\beta = 0.1$, and $\text{data} = 0.5$. The results in (d) and (e) are obtained by averaging over 30 independent simulations. The other parameters are the same as in figure 5 in the main text.

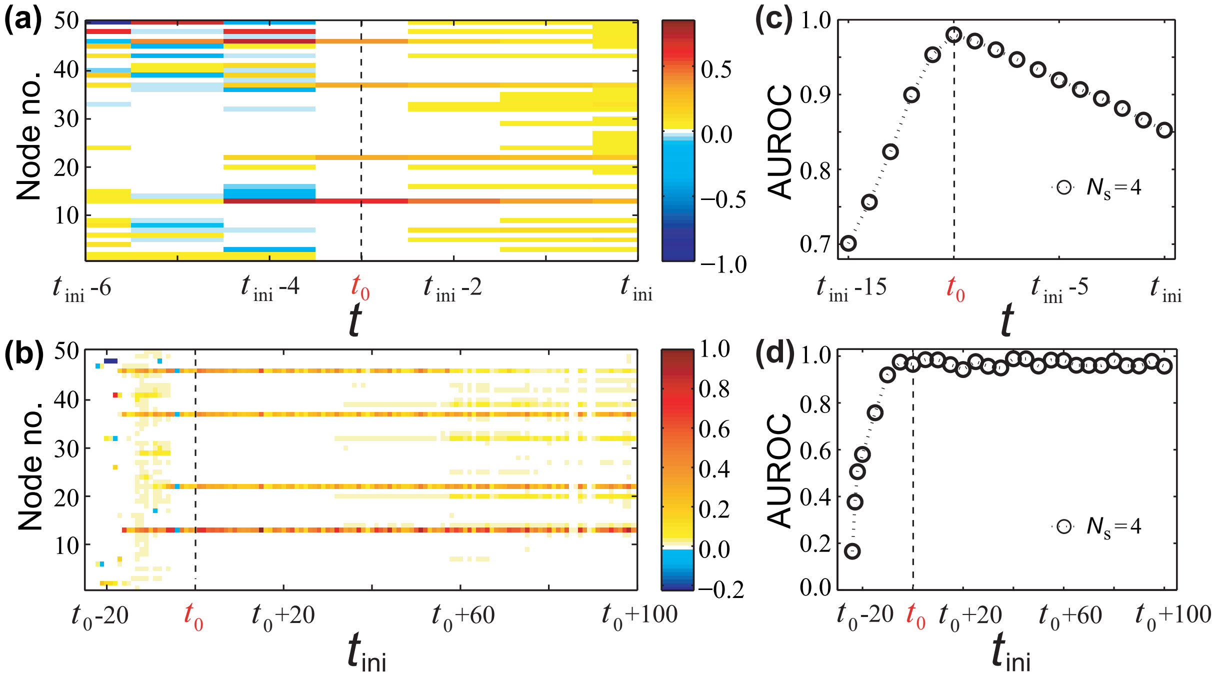

Figure S2. An example of locating sources in undirected weighted SF network with noise. (a) Reconstructed state $x_i(t)$ of each node at time step t, for $t \le t_{\text{ini}}$ and initial observation time t_{ini} ($t_{\text{ini}} \ge t_0$). (b) Reconstructed state $x_i(t)$ of each node for different initial observation time t_{ini} when t_0 is known. (c-d) AUROC as a function of (c) time t ($t \le t_{\text{ini}}$) when the initial observation time is t_{ini} and as a function of (d) initial observation time t_{ini}. Network size is $N = 50$, the average degree is $\langle k \rangle = 4$, and the link weights are uniformly distributed in $(0, 2)$. Four sources are randomly selected and their initial states $x_i(t_0)$ are positive values. We set $\beta = 0.05$, $\sigma = 0.5$, and $\text{data} = 0.5$. The results in (c) and (d) are obtained by averaging over 30 independent simulations. The other parameters are the same as in figure 5 in the main text.

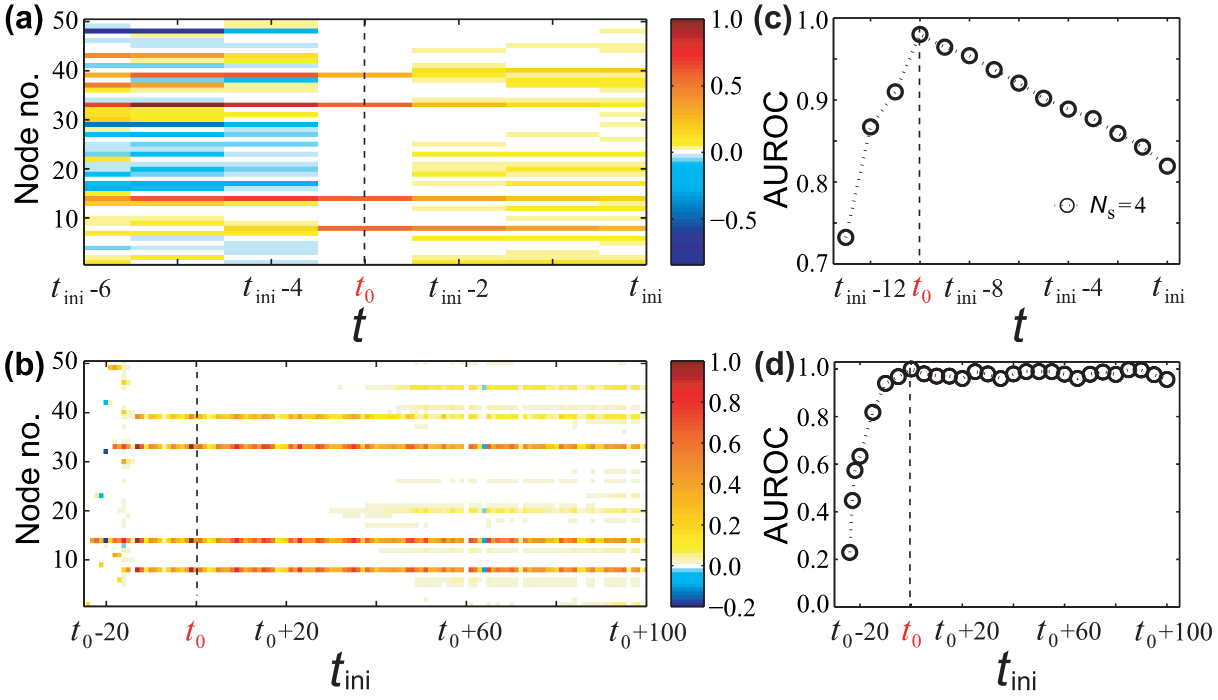

Figure S3. An example of locating sources in undirected weighted ER network with noise. (a) Reconstructed state $x_i(t)$ of each node at time step t for $t \leq t_{\rm ini}$ and for initial observation time $t_{\rm ini}$ ($t_{\rm ini} \geq t_0$). (b) Reconstructed state $x_i(t)$ of each node for different initial observation time $t_{\rm ini}$ and for known t_0. (c-d) AUROC as a function of (c) time t ($t \leq t_{\rm ini}$) when the initial observation time is $t_{\rm ini}$ and as a function of (d) initial observation time $t_{\rm ini}$. Network size is $N = 50$, the average degree is $\langle k \rangle = 4$, and the link weights are uniformly distributed in $(0, 2)$. Four sources are randomly selected and their initial state values $x_i(t_0)$ are positive. We set $\sigma = 0.5$, $\beta = 0.1$, and $\mathrm{data} = 0.5$. The results in (c) and (d) are obtained by averaging over 30 independent simulations. The other parameters are the same as in figure 5 in the main text.

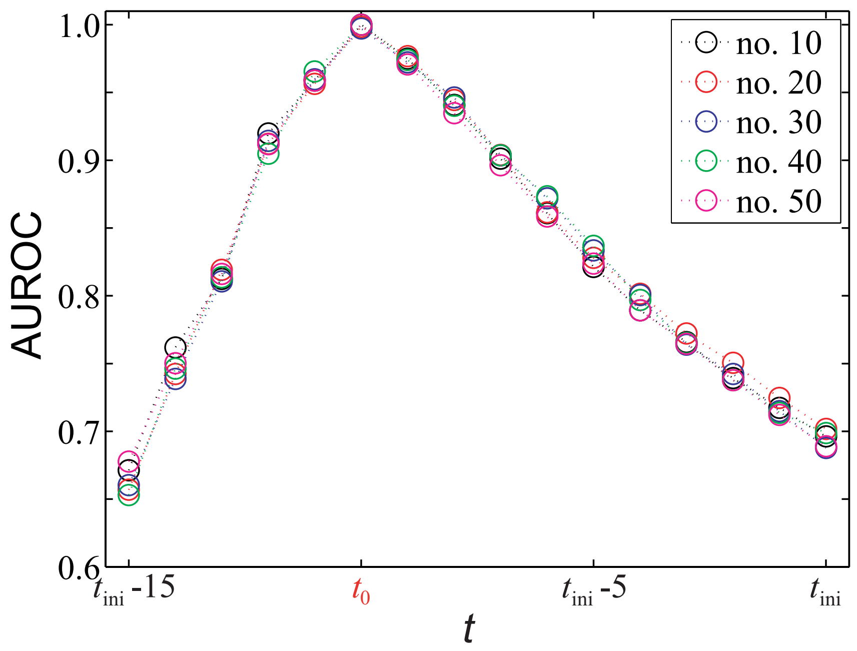

Figure S4. Effect of different choices of messenger node on sources localization and inferring the initial time t_0 in weighted SF networks. AUROC as a function of t ($t \leq t_{\mathrm{ini}}$) with a fixed initial observation time t_{ini}. The actual initial time is t_0, the network size is $N = 50$, and the average degree is $\langle k \rangle = 4$. The link weights are uniformly distributed in $(0, 2)$, $\beta = 0.05$, $\sigma = 0$, and $\mathrm{data} = 0.5$. The number of source N_{s} is 4. The results are obtained by averaging over 100 independent simulations. The other parameters are the same as in figure 5 in the main text.

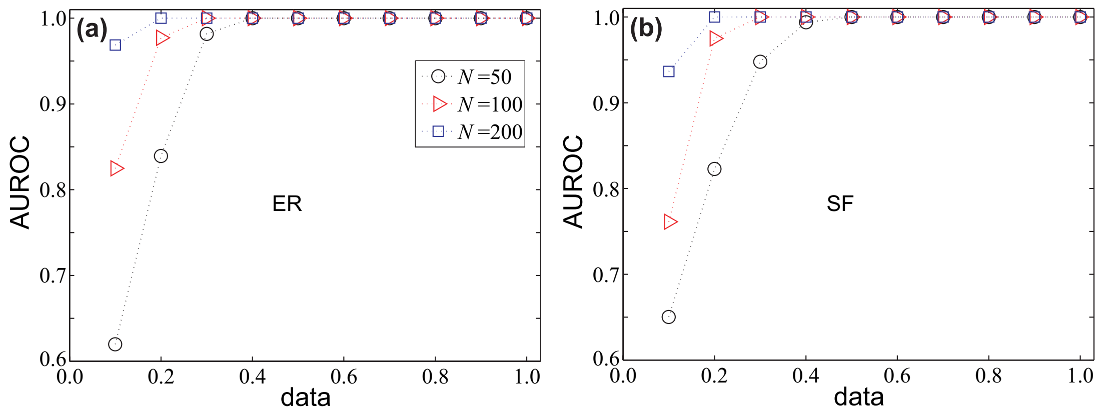

Figure S5. Effect of networks size N on sources localization in networks for different amounts of data. AUROC as a function of data for different network size N for weighted ER networks (a) and for weighted SF networks (b), respectively. The average degree is $\langle k \rangle = 4$ and the link weights are uniformly distributed in $(0, 2)$, $\beta = 0.03$, $\sigma = 0$. The number of source N_{s} is 3. The results are obtained by averaging over 100 independent simulations. The other parameters are the same as in figure 5 in the main text.

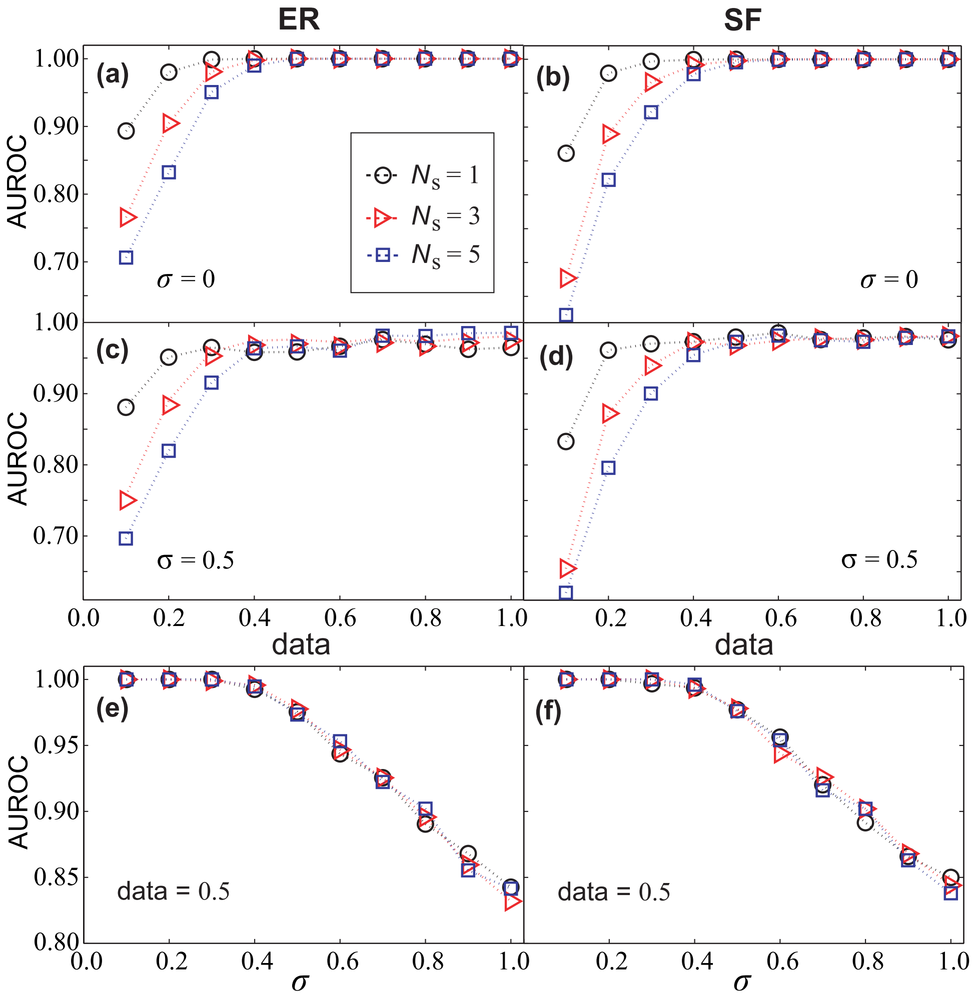

Figure S6. Locating sources in weighted ER and SF networks. (a-b) In absence of noise, AUROC as a function of data for different source number $N_{\rm s}$ for ER and SF networks, respectively. (c-d) The corresponding plots but with noise of amplitude $\sigma = 0.5$. (e-f) For data $= 0.5$, AUROC as a function of σ for different values of $N_{\rm s}$ for ER and SF networks, respectively. The observational noise is modeled as $\hat{\mathbf{y}}(t) = \mathbf{y}(t)[I + \mathcal{N}(\mathbf{0}, \sigma^2 I)]$, where $\mathcal{N}(\mathbf{0}, \sigma^2 I)$ is the Gaussian distribution. The baseline of AUROC is 0.5 (corresponding to random identification). The average degree is $\langle k \rangle = 4$ for both ER and SF networks and the link weights are uniformly distributed in $(0, 2)$. The network size is $N = 50$. We set $\beta = 0.1$ for ER networks and $\beta = 0.05$ for SF networks. The results are obtained by averaging over 1000 independent simulations. The other parameters are the same as in figure 5 in the main text.

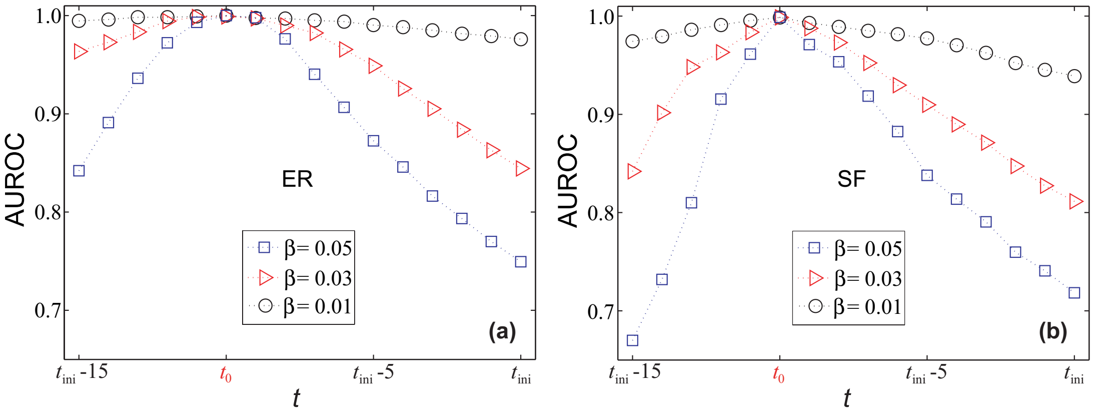

Figure S7. Effect of β on sources localization and inferring the initial time t_0 of sources in networks. AUROC as a function of t ($t \leq t_{\rm ini}$) with a fixed initial observation time $t_{\rm ini}$ for weighted ER networks (a) and weighted SF networks (b), respectively. The true triggering time is t_0. The network size is $N = 50$, and the average degree is $\langle k \rangle = 4$. The link weights are uniformly distributed in $(0, 2)$, $\sigma = 0$, and $\mathrm{data} = 0.5$. The number of source $N_{\rm s}$ is 4. The results are obtained by averaging over 100 independent simulations. The other parameters are the same as in figure 5 in the main text.

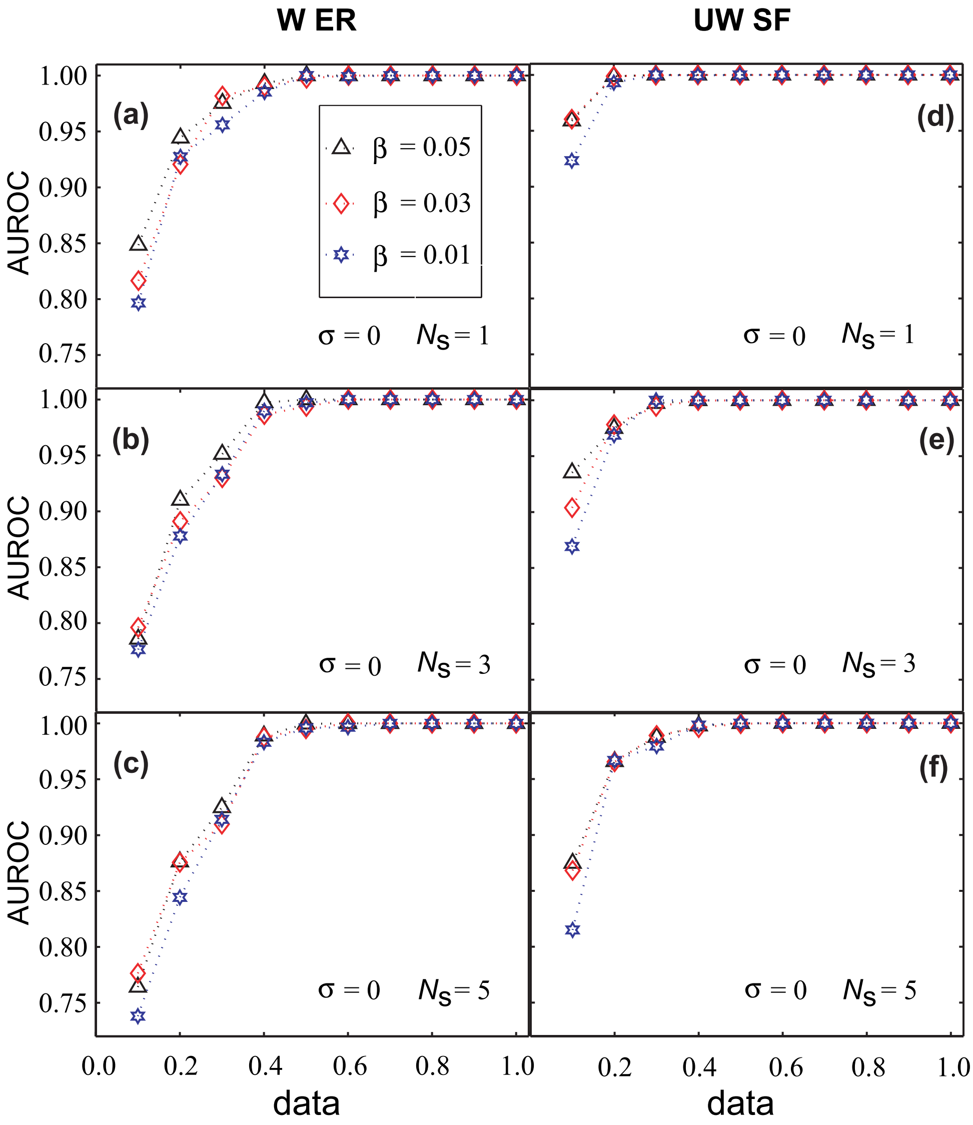

Figure S8. Effect of β on sources localization in networks from different amounts of data. (a-f) AUROC as a function of data for different number N_s of sources for (a-c) weighted ER networks and (d-f) for unweighted SF networks, respectively. For ER networks, $\langle k \rangle = 2$ and for SF networks $\langle k \rangle = 4$. For weighted networks, the link weights are randomly selected from an uniform distribution in the range $(0, 2)$. The network size N is 50 and noise variance $\sigma = 0$. The results are obtained by averaging over 100 independent simulations. The other parameters are the same as in figure 5 in the main text.

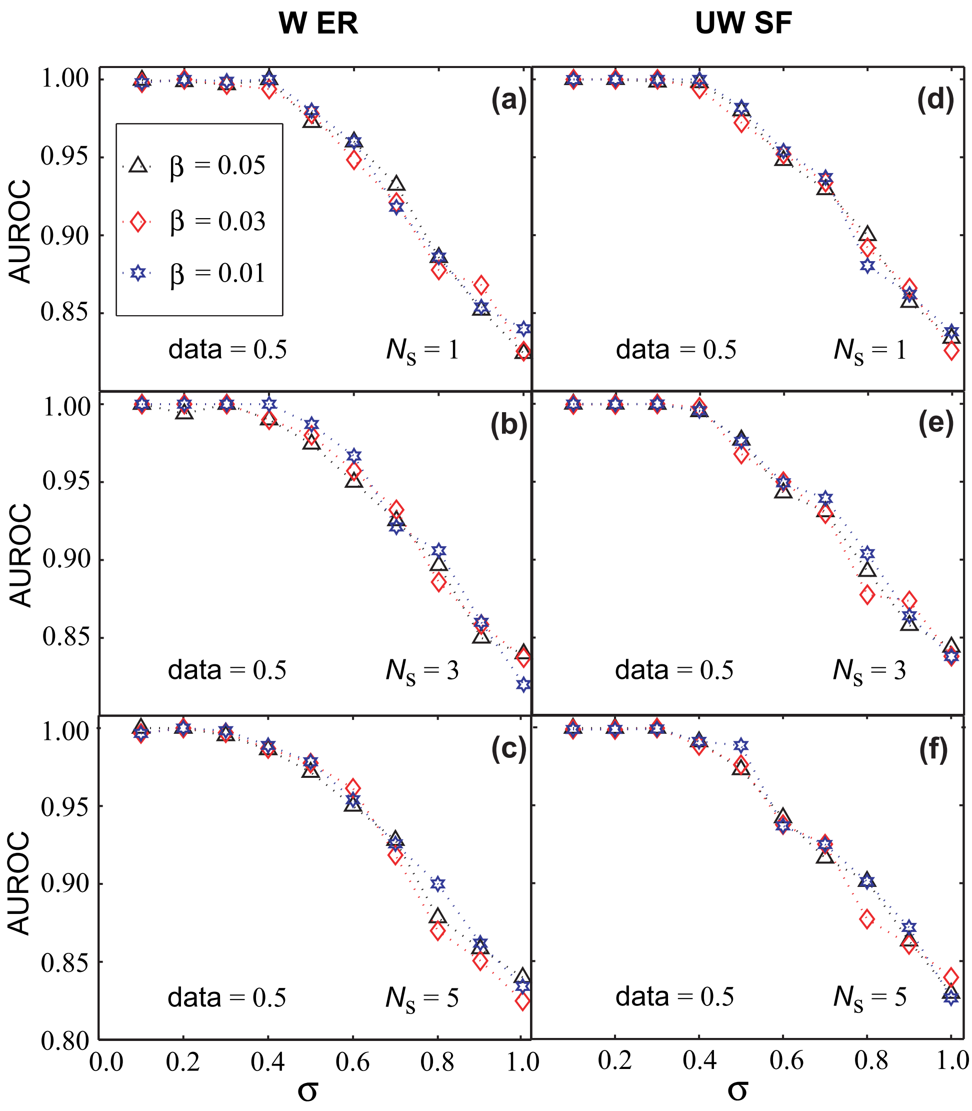

Figure S9. Effect of β on sources localization in networks from noisy data. (a-f) AUROC as a function of noise variance σ for (a-c) weighted ER and (d-f) unweighted SF networks, respectively. The white Gaussian noise is in the form $\hat{\mathbf{y}}(t) = \mathbf{y}(t)[I + \mathcal{N}(\mathbf{0}, \sigma^2 I)]$, where $\mathcal{N}(\mathbf{0}, \sigma^2 I)$ is the Gaussian distribution. data $=$ 0.5 and the results are obtained by averaging over 300 independent simulations. The other parameters are the same as in figure S8.

Supplementary Table

Table S1. Summary of the real networks used in figure 3 in the main text. The quantities N and E denote the network size and the number of links, respectively. UD (D) in the Type column indicates undirected (directed) networks. The structural data of all the networks are available online. The data of Erdös971 can be downloaded via http://vlado.fmf.uni-lj.si/pub/networks/pajek/data/gphs.htm, and the data of USAir can be downloaded via http://vlado.fmf.uni-lj.si/pub/networks/data/map/USAir97.net.

Data sets Name	Type	N	E	Description
ca-GrQc [S8]	UD	5242	14496	collaboration network of Arxiv General Relativity category
ca-HepTh [S8]	UD	9877	25598	collaboration network of Arxiv High Energy Physics Theory category
Erdös971	UD	433	1314	all of Paul Erdös's coauthors and their respective coauthors
dolphin [S9]	UD	62	159	associations between dolphins in a community living off Doubtful Sound
football [S10]	UD	115	613	American football games between Division IA colleges during regular season Fall 2000
Jazz [S11]	UD	198	2742	links of the network of Jazz musicians
Zachary's karate club [S12]	UD	34	78	social network of friendships of a karate club at a US university in the 1970s
Political blogs [S13]	D	1224	19025	hyperlinks between weblogs on US politics in 2005 by Adamic and Glance
Wiki-Vote [S14, 15]	D	7115	103689	all the Wikipedia voting data from the inception of Wikipedia till January 2008
E-mail [S16]	UD	1133	5451	interchanges between members of the Univeristy Rovira i Virgili (Tarragona)
p2p-Gnutella [S17]	D	6301	20777	Gnutella peer-to-peer network on August 8 2002
PGP [S18]	UD	10680	24236	links of the giant component of the network of users of the Pretty-Good-Privacy algorithm for secure information interchange
USAir	UD	332	2126	US Air flights, 1997

References

[S1] Kalman RE. 1959 On the general theory of control systems. *IRE Trans. Automat. Contr.* **4**, 110–110 (doi:10.1109/TAC.1959.1104873).

[S2] Kalman RE. 1963 Mathematical description of linear dynamical systems. *J. Soc. Indus. Appl. Math. Ser. A* **1**, 152–192 (doi:10.1137/0301010).

[S3] Hautus M. 1969 Controllability and observability conditions of linear autonomous systems. *Ned. Akad. Wetenschappen, Proc. Ser. A* **72**, 443 (doi:10.1016/S1385-7258(70)80049-X).

[S4] Yuan Z, Zhao C, Di Z, Wang WX, Lai YC. 2013 Exact controllability of complex networks. *Nat. Commum.* **4**, 1 (doi:10.1038/ncomms3447).

[S5] Zhao C, Wang WX, Liu YY, Slotine JJ. 2015 Intrinsic dynamics induce global symmetry in network controllability. *Sci. Rep.* **5**, 1 (doi:10.1038/srep08422).

[S6] Erdös P, Rényi A. 1960 On the evolution of random graphs. *Publ. Math. Inst. Hungar. Acad. Sci.* **5**, 17–61 (doi:10.2307/1999405).

[S7] Liu YY, Slotine JJ, Barabási AL. 2011 Controllability of complex networks. *Nature* **473**, 167–173 (doi:10.1038/nature10011).

[S8] Leskovec J, Kleinberg J, Faloutsos C. 2007 Graph evolution: Densification and shrinking diameters. *ACM TKDD* **1**,2 (doi:10.1145/1217299.1217301).

[S9] Lusseau D, *et al.,* 2003 The bottlenose dolphin community of doubtful sound features a large proportion of long-lasting associations. *Behav. Ecol. Sociobiol.* **54**, 396–405 (doi:10.1007/s00265-003-0651-y).

[S10] Girvan M, Newman ME. 2002 Community structure in social and biological networks. *Proc. Natl Acad. Sci. U.S.A.* **99**, 7821–7826 (doi: 10.1073/pnas.122653799).

[S11] Gleiser PM, Danon L. 2003 Community structure in jazz. *Adv. Complex Syst.* **6**, 565–573 (doi:10.1142/S0219525903001067).

[S12] Zachary WW. 1977 An information flow model for conflict and fission in small groups. *J. Anthropol. Res.* **33**, 452–473 (doi:10.1086/jar.33.4.3629752).

[S13] Adamic LA, and Glance N, 2005 The political blogosphere and the 2004 us election: divided they blog. In *Proceedings of the 3rd international workshop on Link discovery, ACM* 36–43 (doi:10.1145/1134271.1134277).

[S14] Leskovec J, Huttenlocher D, Kleinberg J. 2010 Signed networks in social media. In *Proceedings of the SIGCHI Conference on Human Factors in Computing Systems, ACM* 1361–1370 (doi:10.1145/1753326.1753532).

[S15] Leskovec J, Huttenlocher D, Kleinberg J. 2010 Predicting positive and negative links in online social networks. In *Proceedings of the 19th international conference on World wide web, ACM* 641–650 (doi:10.1145/1772690.1772756).

[S16] Guimera R, Danon L, Diaz-Guilera A, Giralt F, Arenas A. 2003 Self-similar community structure in a network of human interactions. *Phys. Rev. E* **68**, 065103 (doi:10.1103/PhysRevE.68.065103).

[S17] Ripeanu M, Foster I, Iamnitchi A. 2002 Mapping the gnutella network: Properties of large-scale peer-to-peer systems and implications for system design. In *IEEE Internet Computing Journal* 1-12 (doi:10.1109/4236.978369).

[S18] Boguñá M, Pastor-Satorras R, Díaz-Guilera A, Arenas A. 2004 Models of social networks based on social distance attachment. *Phys. Rev. E* **70**, 056122 (doi:10.1103/PhysRevE.70.056122).
